# Hsa_circ_0136666 stimulates gastric cancer progression and tumor immune escape by regulating the miR-375/PRKDC Axis and PD-L1 phosphorylation

**DOI:** 10.1186/s12943-023-01883-y

**Published:** 2023-12-13

**Authors:** Zhenyan Miao, Jifei Li, Yu Wang, Mingqin Shi, Xiao Gu, Xuanqi Zhang, Fang Wei, Xinying Tang, Lufeng Zheng, Yingying Xing

**Affiliations:** 1 School of Life Science and Technology, China Pharmaceutical University, Nanjing, 210009 People’s Republic of China; 2 Jiangsu Key Laboratory of Carcinogenesis and Intervention, China Pharmaceutical University, Nanjing, 210009 People’s Republic of China; 3 State Key Laboratory of Natural Medicines, China Pharmaceutical University, Nanjing, 210009 People’s Republic of China; 4 Center for New Drug Safety Evaluation and Research, China Pharmaceutical University, Nanjing, 210009 People’s Republic of China

**Keywords:** Circular RNA, miR-375, PD-L1, Immune escape, Gastric cancer

## Abstract

**Background:**

Targeted drugs are not quite effective for prolonging the survival of patients with gastric cancer due to off-target effects as well as tumor immune escape mechanisms. Circular RNAs widely exist in tumor regions as biomarkers and can be developed as effective drug targets.

**Methods:**

Western blot, QRT-PCR, fluorescence in situ hybridization, and flow cytometry were used to investigate the function of hsa_circ_0136666 in promoting the proliferation of gastric cancer cells. Tissue immunofluorescence, enzyme-linked immunosorbent assay (ELISA), as well as flow cytometric analysis, was conducted to explore the process of tumor immune evasion in tumor-bearing mice. The differences of circRNA expression in clinical samples were analyzed through tissue microarray FISH. The effect of siRNA on improving the efficacy of anti-PDL1 drugs and suppressing the immune microenvironment was evaluated by the coadministration model.

**Results:**

We demonstrated that hsa_circ_0136666 was widely and highly expressed in gastric cancer tissues and cells. Functionally, hsa_circ_0136666 promoted gastric cancer tumor proliferation and tumor microenvironment formation, leading to tumorigenesis immune escape, and this effect was dependent on CD8 + T cells. Mechanistically, we confirmed that hsa_circ_0136666 competitively upregulated PRKDC expression by sponging miR-375-3p, regulating immune checkpoint proteins, prompting phosphorylation of PD-L1 to preventing its degradation, driving PD-L1 aggregation and suppressing immune function, thereby impairing cancer immune responses. In terms of application, we found that LNP-siRNA effectively improved anti-PDL1 drug efficacy and inhibited immune escape.

**Conclusion:**

Our results reveal an oncogenic role played by hsa_circ_0136666 in gastric cancer, driving PD-L1 phosphorylation via the miR-375/PRKDC signaling axis, prompting immune escape. This work proposes a completely new pathogenic mechanism of gastric cancer, uncovers a novel role for hsa_circ_0136666 as an immune target, and provides a rationale for enhancing the efficacy of anti-PD-L1 therapy for gastric cancer.

**Supplementary Information:**

The online version contains supplementary material available at 10.1186/s12943-023-01883-y.

## Introduction

Gastric cancer is a significant public health problem worldwide, with high incidence and mortality rates in many countries. The disease is responsible for over 700,000 deaths annually, making it the third leading cause of cancer-related mortality worldwide [1]. Symptoms of stomach cancer are often present only in advanced stages of the disease, making early detection difficult [2, 3]. Due to the limitations imposed by patient health status, tumor drug resistance, and side effects, current treatment options for gastric cancer have not been able to meet expectations [4]. Therefore, efficient early diagnostic biomarkers and potent therapeutic strategies for GC are urgently needed.

Circular RNAs (circRNAs) are abundant and highly conserved noncoding RNAs (ncRNAs) and are characterized by covalent closed-loop structures [5, 6]. The key physiological functions of circRNAs include miRNA sponges, transcriptional regulators, and binding to RNA-binding proteins. And circRNAs play important biological functions by regulating protein functions or encoding recessive peptides [7]. In recent years, circRNAs are associated with diseases such as diabetes, neurological diseases, cardiovascular diseases, and cancers [8, 9]. It has been shown that circRNAs may play physiological roles during gastric carcinogenesis [10, 11]. circRNAs can interact with microRNAs (miRNAs), thereby regulating miRNA-targeted gene expression by competitively binding to miRNA response elements [12].

MicroRNA (miRNA), similar to circRNA, is a widely abundant non-coding RNA that can bind to mRNA to degrade target genes and exert its biological function [13]. Our previous research found that miR-375 is down-regulated in gastric cancer and plays a suppressive role in gastric cancer [14, 15].

When discussing the starting point of our research, we chose to focus on circPRKDC. Since PRKDC is the catalytic subunit of DNA-dependent protein kinase (DNA-PK), which plays a crucial role in DNA double-strand break repair and recombination [16]. PRKDC also functions as an important protein kinase in cells, phosphorylating multiple proteins to regulate their activity [17]. The immune checkpoint protein is regulated by various post-translational modifications. For example, the PD-L1 protein is regulated by phosphorylation, palmitoylation and ubiquitination [18–20], which plays a promoting role in the development of tumors [21, 22]. These post-translational modifications can affect the properties of PDL1 and regulate its function.

In our study, we found that hsa_circ_0136666 is highly expressed in gastric cancer and interacts with miR-375 in a sponge-like manner. Through miR-375, hsa_circ_0136666 promotes the expression of PRKDC, which can phosphorylate PDL1 and enhance its stability, thereby promoting gastric cancer progression and immune escape.

## Results

### Hsa_circ_0136666 has intersection with miR-375, which positively associated with gastric cancer growth

**Fig. 1:**
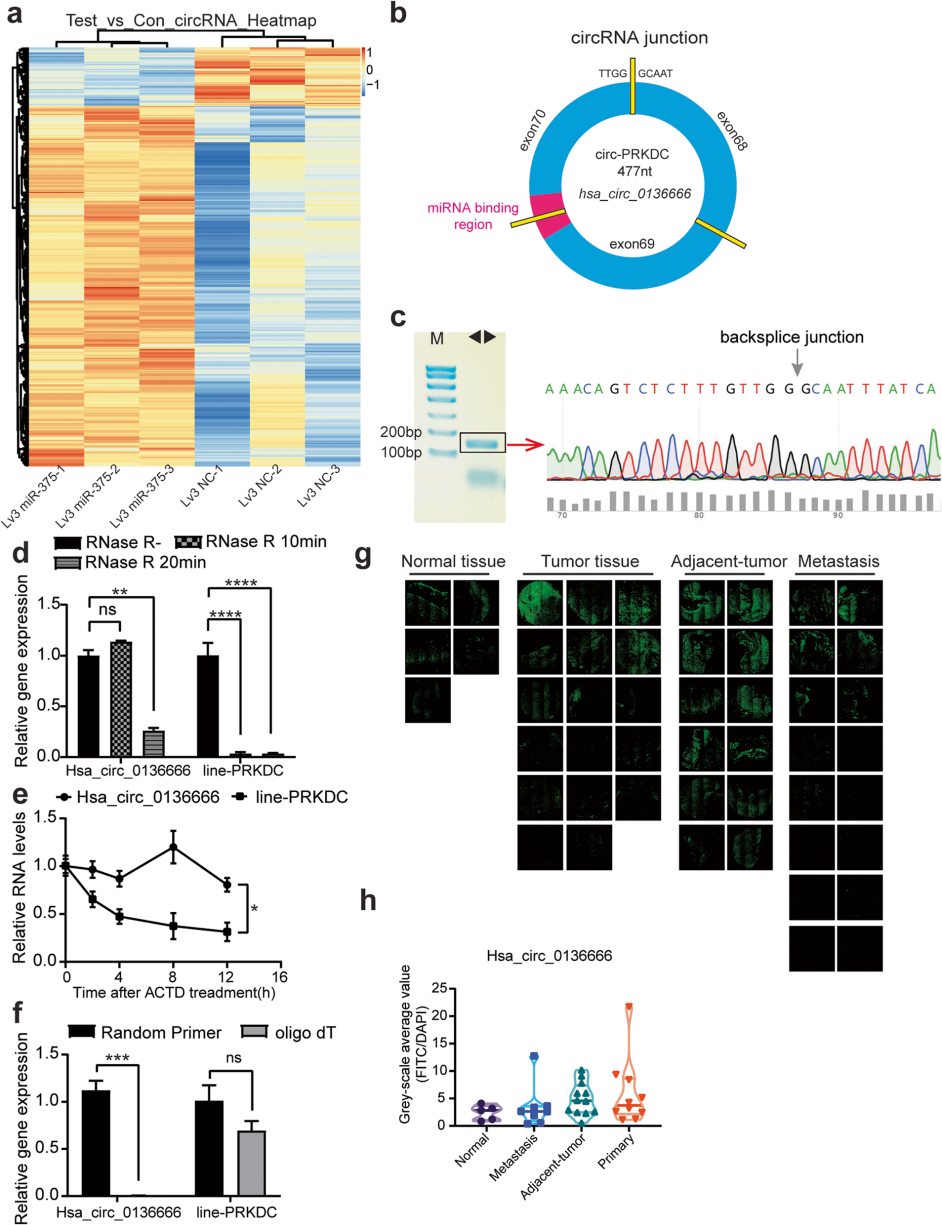
Hsa_circ_0136666 has intersection with miR-375, which positively associated with gastric cancer growth. **a** Hsa_circ_0136666 is enriched in differentially expressed genes in gene chip. **b** Schematic diagram of hsa_circ_0136666 biosynthesis. **c** Divergent primers were used for amplification, and PCR products were Sanger sequenced. **d** Hsa_circ_0136666 and linear-PRKDC gene expression abundance were detected under RNase digestion, *n* = 3. **e** The natural degradation rate of hsa_circ_0136666 and linear-PRKDC gene was detected under Actinomycin D treatment, *n* = 3. **f** Hsa_circ_0136666 and linear PRKDC genes were amplified using oligo dT and random primers, *n* = 3. **g** The expression of hsa_circ_0136666 in gastric cancer tissue sections was presented by FITC fluorescence. **h** Differential expression of hsa_circ_0136666 in tissues. Data are presented as the mean ± SD, Student's t-test was used. *P < 0.05, **P < 0.01, ***P < 0.001, ****P < 0.0001

In our previous study, we found that miR-375 could inhibit gastric cancer progression. We discovered that overexpression of miR-375 caused aberrant changes in long non-coding RNAs including circRNAs. It is speculated that the expression of endogenous miR-375 may be regulated by upstream molecules. To identify potential circRNAs that acts as a miR-375 sponge, we screened out the differentially expressed circRNAs through Gene Expression Omnibus (GEO) repository (GSE147698). In total, 1466 circular RNAs were differentially expressed (*P* < 0.05, FC > 1.2), of which 1262 were upregulated genes (Fig. 1a). In parallel, we screened for circRNAs candidates (among these 1262 circRNAs) with binding sites to miR-375 from the ENCORI online database(rnasysu.com/encori/index.php). We learned that both circPRKDC and PRKDC have binding sites with miR-375. We also found the only case of circPRKDC with a binding site to miR-375, namely hsa_circ_0136666. The hsa_circ_0136666 isoform located at chr8: 48,715,866–48,730,122 in the human genome, was derived from the circularization of exon-68 to exon-70 of the PRKDC gene. The exons were joined end-to-end by back splicing to form a circular RNA 477nt in length (Fig. 1b). Divergent primers were designed to verify the back-splice site, and the PCR amplification products were sequenced by Sanger to confirm the existence of hsa_circ_0136666 in gastric cancer cells (Fig. 1c).

To further confirm the ring structure, we conducted an RNase R enzyme digestion experiment. After enzymatic digestion by RNase R, the circular RNA was less degraded and the linear RNA (PRKDC) was mostly degraded (Fig. 1d). Hsa_circ_0136666 also had a lower natural degradation rate than PRKDC after the addition of actinomycin D (Fig. 1e). We then verified hsa_circ_0136666 with oligo dT, for that hsa_circ_0136666 could not be amplified by oligo dT. To this point, we have confirmed that hsa_circ_0136666 has a circular structure with greater stability than the parental gene (Fig. 1f).

We made use of fluorescent probes to detect the distribution of hsa_circ_0136666 in gastric cancer tissues, the results showed that hsa_circ_0136666 was widely expressed in different stages of gastric cancer (Fig. 1g). Compared with normal tissues, the expression level was higher in carcinoma in situ tissues, followed by paracancerous tissues and metastases (Fig. 1h), which showed that hsa_circ_0136666 played a tremendous part in the development of gastric cancer. According to the analysis of clinical patients with gastric cancer in TCGA database, high expression of miR-375 prolonged the survival of patients, and low expression had a trend of short survival (Supplementary Fig. 1a). The expression of PRKDC was negatively correlated with the survival time of patients (Supplementary Fig. 1b).

In summary, hsa_circ_0136666 had a ring-like structure that was not easily degraded, which was highly expressed in gastric cancer tissues. We speculated that hsa_circ_0136666 was implicated in regulatory mechanisms during gastric cancer progression and was inseparable from cancer progression.

### Hsa_circ_0136666 promotes tumor proliferation and tumor immune escape

**Fig. 2:**
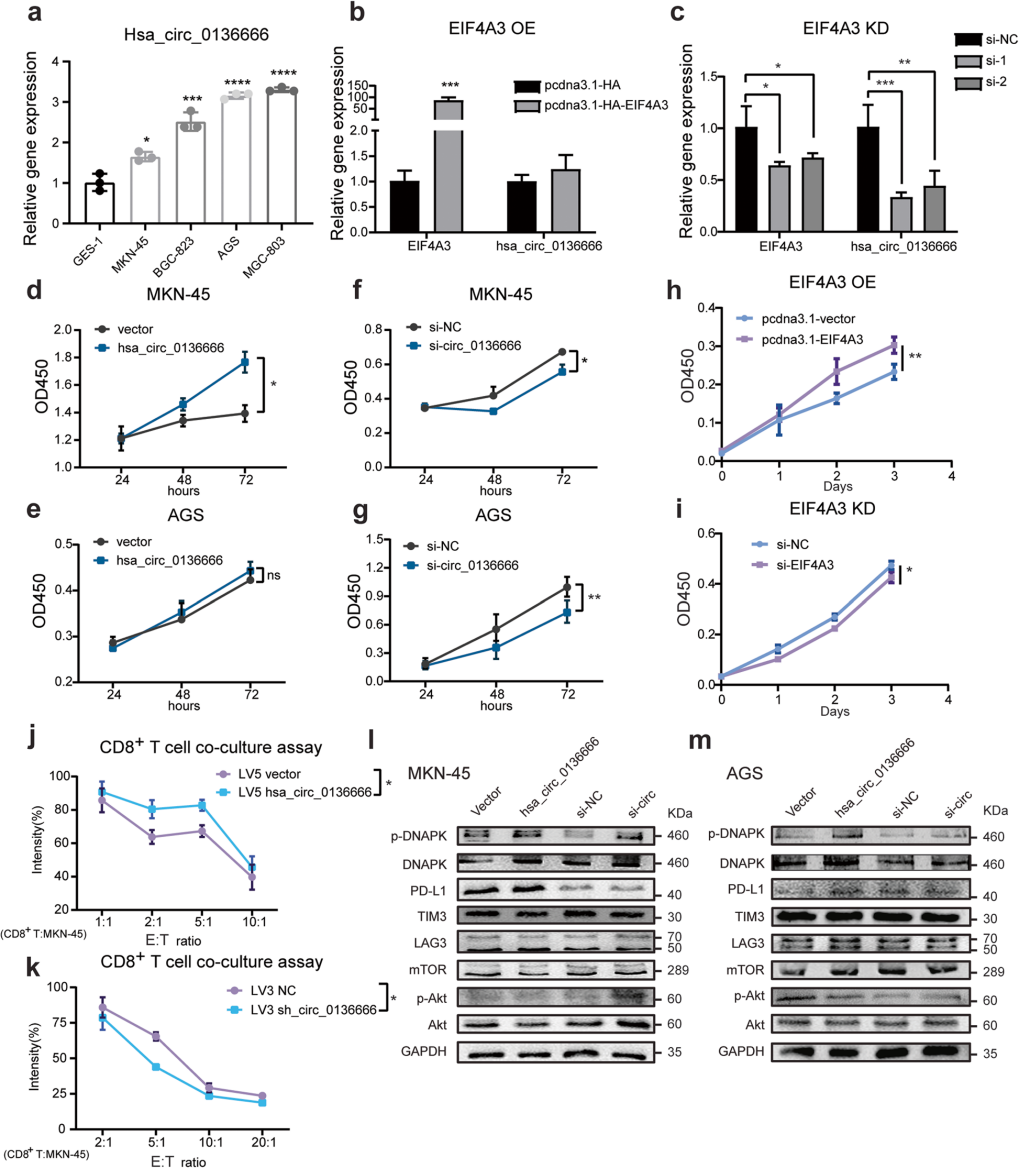
Hsa_circ_0136666 promotes tumor proliferation and tumor immune escape. **a** Expression of hsa_circ_0136666 in various gastric cancer cell lines, compared to GES-1. **b** The effect of the overexpression of EIF4A3 on the expression abundance of hsa_circ_0136666. **c** The effect of the knockdown of EIF4A3 on the expression abundance of hsa_circ_0136666. **d** Overexpression of hsa_circ_0136666 promoted cell proliferation in MKN-45 cells. **e** Overexpression of hsa_circ_0136666 promoted cell proliferation in AGS cells. **f** Knockdown of hsa_circ_0136666 inhibited cell proliferation in MKN-45 cells. **g** Knockdown of hsa_circ_0136666 inhibited cell proliferation in AGS cells. **h** Overexpression of EIF4A3 promoted cell proliferation in MKN-45 cells. **i** Knockdown of EIF4A3 inhibited cell proliferation in MKN-45 cells. **j** Image of survival rate of overexpressing hsa_circ_0136666 tumor cells co-cultured with CD8^+^ T cells, *n* = 6. **k** Image of survival rate of knockdown hsa_circ_0136666 tumor cells co-cultured with CD8^+^ T cells, *n* = 6. **l** Western blot was used to detect immune checkpoint proteins and PI3 signaling pathway proteins in MKN-45 cells. **m** Western blot was used to detect immune checkpoint proteins and PI3 signaling pathway proteins in AGS cells. Data are presented as the mean ± SD, *n* = 3 if not mentioned. Student's t-test was used. *P < 0.05, **P < 0.01, ***P < 0.001, ****P < 0.0001

We have found that hsa_circ_0136666 was ubiquitously expressed in different gastric cancer tissues, and this condition could also be found in cell lines. The abundance of hsa_circ_0136666 was generally upregulated in tumor cell lines compared with normal gastric epithelium cell GES-1 (Fig. 2a). Thus, hsa_circ_0136666 expression is region specific and highly expressed in gastric cancer.

According to a previous literature report [23], EIF4A3 is a core component of the exon junction complex and plays an essential role in pre-mRNA splicing, we learned that EIF4A3 dominated the biosynthesis and back-splicing process of circRNAs. Circular RNA interactome (https://circinteractome.nia.nih.gov/index.html) showed that there were four binding sites of EIF4A3 in the upstream and downstream of hsa_circ_0136666 pre-mRNA. QRT-PCR experiments showed that overexpression of EIF4A3 could promote hsa_circ_0136666 expression, while knockdown of EIF4A3 could suppress the expression of hsa_circ_0136666 in gastric cancer cells (Fig. 2b-c).

CCK-8 experiments were performed to find that hsa_circ_0136666 overexpression promoted cancer cell proliferation (Fig. 2d-e), while knockdown of hsa_circ_0136666 using siRNA resulted in cell growth inhibition (Fig. 2f-g). Hsa_circ_0136666 is of vital importance during tumor growth. We also noticed that the growth rate of gastric cancer cells was continuously promoted by the presence of EIF4A3 (Fig. 2h). Conversely, the cell growth rate was inhibited with the knockdown of EIF4A3 (Fig. 2i).

GO analysis (Biological_Process section) related to circRNAs in GSE147698 data showed that genes negatively correlated with miR-375 were mainly enriched in signal transduction, immune response, and inflammatory response (Supplementary Fig. 2a). KEGG pathway analysis also enriched in the T cell receptor signaling pathway (Supplementary Fig. 2b). It had been reported that tumor patients with PRKDC mutation tended to have a more robust response to immunotherapy, illustrating that PRKDC contributed to malignancy by suppressing immunity [24]. We explored how hsa_circ_0136666 regulated tumor growth via immune pathways. Since CD8^+^ T cells are recognized as the main effector cells of cell immunity which kill cancer cells by releasing perforin, granzyme B and IFN-γ. We found that cancer cells overexpressing hsa_circ_0136666 had an increased survival rate under coculture with isolated and reactivated CD8^+^ T cells, which was reversed by the knockdown of hsa_circ_0136666 (Fig. 2j-k). As in our conjecture, hsa_circ_0136666 contributed to tumor cells resistance to the anti-tumor immunity.

Immune checkpoint blockade has the broadest impact in different types of cancer immunotherapy, with some antibodies against the cytotoxic T lymphocyte antigen 4 (CTLA4) or programmed cell death 1 (PD1)—PD1 ligand 1 (PD-L1) axis being approved for many different cancers. Interferon-γ response with activation of the PD-L1 pathway, upregulation of other immune related pathways, along with downregulation of Transforming growth factor-β may contribute to improved treatment of patients with PRKDC mutations [25]. Thus we began to study immune-related molecules and found that hsa_circ_0136666 was closely related to immune checkpoint molecules. Hsa_circ_0136666 could significantly increase the amount of immune checkpoint protein expression, especially PD-L1 (Fig. 2l and Supplementary Fig. 4e-f), while the knockdown of hsa_circ_0136666 using siRNA decreased PD-L1 expression (Fig. 2m and Supplementary Fig. 4g-h). During the experiment, we also found that hsa_circ_0136666 had little effect on the level of immune checkpoint genes, and hsa_circ_0136666 knockdown may lead to downregulation of gene expression. This also suggested that hsa_circ_0136666 may not regulate immunity through mRNA levels (Supplementary Fig. 4a-d).

Therefore, hsa_circ_0136666 could effectively promote tumor cells proliferation in vitro without being detected by immune cells, and the biosynthesis of hsa_circ_0136666 is also regulated by EIF4A3.

### Hsa_circ_0136666 regulates antitumor immune responses driven toward immune escape

**Fig. 3:**
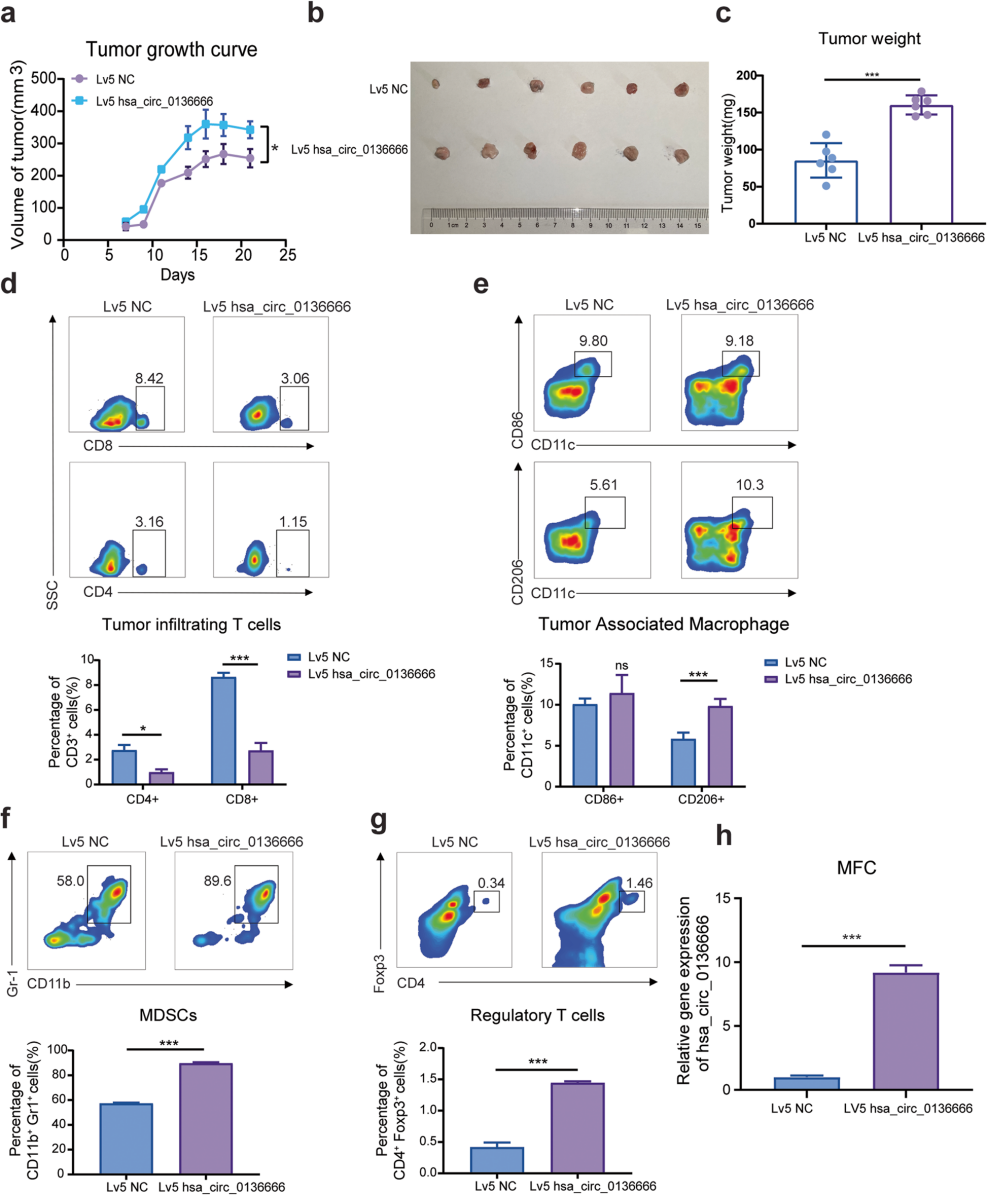
Hsa_circ_0136666 regulates antitumor immune responses driven toward immune escape. **a** Tumor growth curve of tumor bearing mice with Lv5 MFC cells. **b** Original photo of mouse tumor. **c** Comparison of tumor weight in mice. **d** Differences in the proportion of tumor infiltrating T lymphocytes. **e** Differences in the proportion of tumor associated macrophages. **f** Differences in the proportion of tumor infiltrating MDSC cells. **g** Differences in the proportion of tumor infiltrating regulatory T cells. Data are presented as the mean ± SD, *n* = 6, Student's t-test was used. *P < 0.05, ***P < 0.001. **h** The overexpression of hsa_circ_0136666 in MFC. ***P < 0.001

Murine gastric cancer cells were utilized to construct a mouse model for homologous tumor transplantation. Tumor-bearing C57BL/c mice inoculated with MFC-hsa_circ_0136666 cells had a faster tumor growth rate (Fig. 3a) and larger tumor weight (Fig. 3b-c). In the BALB/c nude tumor-bearing mice model, we reached a similar conclusion (Supplementary Fig. 3a-b). This revealed that the humanized hsa_circ_0136666 led to an exacerbation of cancer during tumor growth progression in mice. The tumor immune microenvironment is currently often considered an inhibitor and brake on antitumor therapies, with many immunosuppressive cells and cytokines playing important functions therein. We next explored whether hsa_circ_0136666 was involved in immune cell trafficking in the TME and we focused on effector T cells, Treg cells, M2 like macrophages, and myeloid-derived suppressor cells (MDSCs). We found that the total amount of tumor-infiltrating T cells significantly decreased with overexpression of hsa_circ_0136666 and tumor-associated macrophages were polarized toward the M2 phase during the resistance phase of antitumor immunity (Fig. 3d-e). In addition, there was a trend toward significant increases in both MDSCs and Treg cells with the presence of hsa_circ_0136666 over-expressing (Fig. 3f-g). We validated the reliability of the hsa_circ_0136666 overexpression MFC cell model, which showed that hsa_circ_0136666 can be stably overexpressed in MFC (Fig. 3h). Various indications suggested that tumor growth proceeded toward malignancy in the presence of hsa_circ_0136666.

Previous studies have learned that cytokines also play a key role in cellular immunity. For example, TGF-β shapes the tumor microenvironment and suppresses anti-tumor immunity by limiting T cell infiltration [26]. PD-1 mAb treatment induces IFN-γ-induced tumor cell YAP aggregation, which enhances key immunosuppressive target genes by forming a transcriptional hub. The expression of IFN-γ can mediate the adaptive resistance of tumor cells [27]. The IL-6/STAT3 axis can simultaneously promote the expansion of immunosuppressive cells or change the balance of T cell subsets [28], and targeting IL-6 can enhance the anti-PD-L1 healing effect [20]. As we found, IFN-γ and IL-6 showed an upward trend in the tumor tissues overexpressing hsa_circ_0136666, while TGF-β had no significant change. However, these pro-oncogenic factors showed a down-regulation trend in knockdown tissues (Supplementary Fig. 3c-e). Immunohistochemical analysis of pathological sections from the tumorigenic area revealed severer local deterioration and greater PD-L1 expression (Supplementary Fig. 3f). Above, we found that hsa_circ_0136666 promoted rapid tumor growth and immune microenvironment formation in vivo.

### Hsa_circ_0136666 regulates gastric cancer progression via miR-375 sponge

**Fig. 4:**
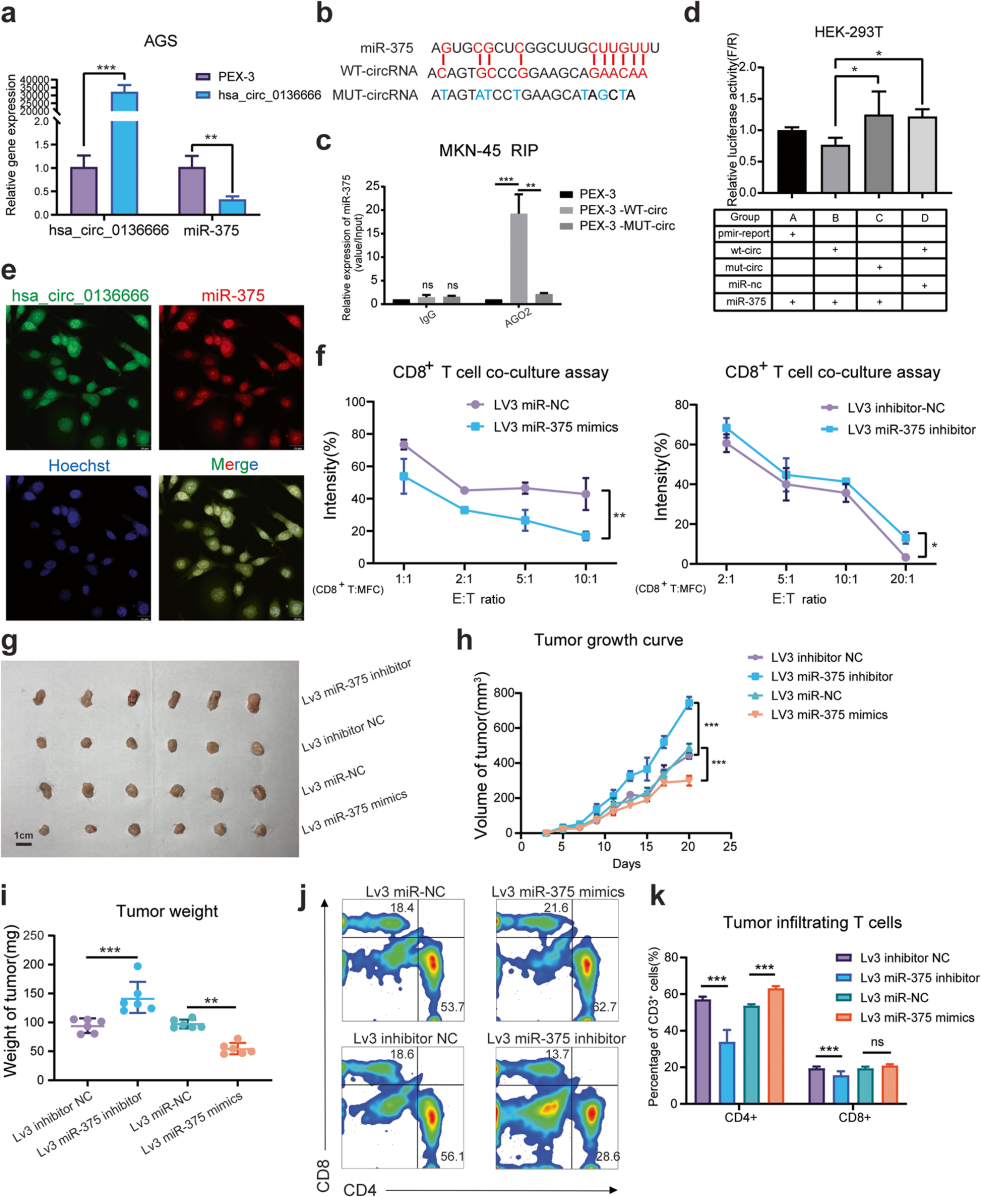
Hsa_circ_0136666 regulates gastric cancer progression via miR-375 sponge. **a** The negative correlation between the relative expression of hsa_circ_0136666 and miR-375, *n* = 3. **b** MiR-375 has complementary base pairing sites with wild-type hsa_circ_0136666. **c** RIP experiment was used to verify that hsa_circ_0136666 exerts sponge function, *n* = 3. **d** Dual luciferase reporter assay was used to verify the interaction between hsa_circ_0136666 and miR-375, *n* = 6. **e** RNA in situ hybridization was implemented to verify the colocalization of hsa_circ_0136666 with miR-375. **f** Image of survival rate of overexpressing and knockdown hsa_circ_0136666 tumor cells co-cultured with CD8^+^ T cells, *n* = 6. **g** Original photo of tumor in Lv3 miR-375 tumor bearing mice. **h** Tumor growth curve of Lv3 miR-375 tumor bearing mice, *n* = 6. **i** Comparison of tumor weight in mice, *n* = 6. **j** Differential distribution of tumor infiltrating T lymphocytes in different groups of Lv3 tumor bearing mice, *n* = 6. **k** Quantitative distribution of tumor infiltrating T lymphocytes in different groups, *n* = 6. Data are presented as the mean ± SD, Student's t-test was used. *P < 0.05, **P < 0.01, ***P < 0.001

Our previous studies have confirmed that miR-375 can inhibit gastric carcinogenesis induced by *H. pylori* and that *H. pylori* can affect the levels of inflammatory factors and the differentiation of immune cells in the stomach by downregulating miR-375 expression. As we previously predicted, hsa_circ_0136666 was negatively correlated with miR-375 expression (Fig. 4a). We utilized the online website starBase v2.0 (http://starbase.sysu.edu.cn/starbase2) to search for the hsa_circ_0136666 and miR-375 binding regions (Fig. 4b). RIP assays were used to corroborate intermesh. Argonaute 2(AGO2) was a key component of the RISC complex, and anti-AGO2 antibody can be used to pull down miR-375 bound to Wild-Type hsa_circ_0136666 (Fig. 4c and Supplementary Fig. 5e). The dual luciferase reporter assay also confirmed that hsa_circ_0136666 could bind to miR-375, and the combination disappeared after mutating the binding site (Fig. 4d).

To explore whether hsa_circ_0136666 exerts a cancer-promoting effect by sponging miR-375. By taking advantage of RNA fluorescence in situ hybridization, we confirmed that hsa_circ_0136666 mainly exists in the cytoplasm, and has almost identical distribution with miR-375 (Fig. 4e). In addition, miR-375 had the function of regulating tumor immunity. Cancer cells transfected miR-375 mimics had a decreased survival rate under coculture with isolated and reactivated CD8^+^ T cells, while cancer cells transfected miR-375 inhibitor had an increased rate (Fig. 4f). Meanwhile, we validated the effect of miR-375 on tumor growth in C57BL/c tumor bearing mice (Fig. 4g). The tumor growth rate and tumor weight were also reduced by miR-375 overexpression, miR-375 inhibitor significantly promoted tumor growth and increased tumor weight (Fig. 4h-i). When we examined T cells viability in the tumor region, we found that T cells expansion were significantly increased with miR-375 overexpression, both CD4^+^ T and CD8^+^ T cells showed an increasing trend (Fig. 4j). However, the phenomenon of T-cell proliferation was reversed in the miR-375 inhibitor group (Fig. 4k). We reached the conclusion that hsa_circ_0136666 was acted as a miR-375 sponge to inhibit miR-375 from playing a physiological role.

### Hsa_circ_0136666 regulates immune responses through the miR-375/PRKDC signaling axis

**Fig. 5:**
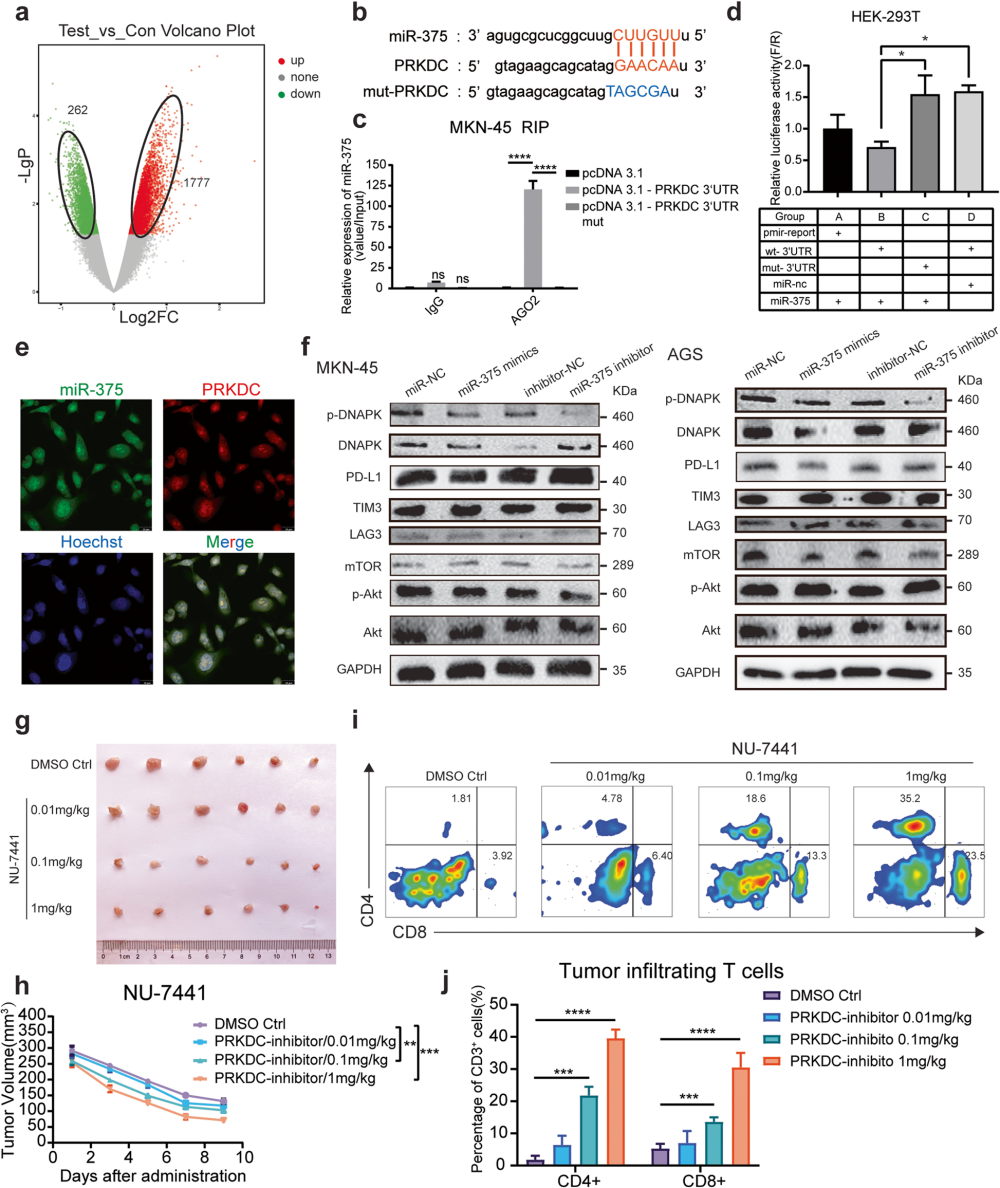
Hsa_circ_0136666 regulates immune responses through the miR-375/PRKDC signaling axis. **a** The gene chip overexpressing miR-375 enriched 262 downregulated genes and 1777 upregulated genes. **b** MiR-375 has complementary base pairing sites with wild-type PRKDC mRNA 3’UTR. **c** RIP experiment was used to verify the combination of miR-375 and PRKDC, *n* = 3. **d** Dual luciferase reporter assay was used to verify the interaction between PRKDC 3’UTR and miR-375, *n* = 6. **e** RNA in situ hybridization was implemented to verify the colocalization of PRKDC mRNA 3’UTR with miR-375. **f** Western blot was used to detect immune checkpoint proteins and PI3 signaling pathway proteins in MKN-45 cells while miR-375 overexpressing. **g** Original photo of tumor in tumor bearing mice under NU-7441 treatment. **h** Tumor growth curve of tumor bearing mice under NU-7441 treatment, *n* = 6. **i** Differential distribution of tumor infiltrating T lymphocytes in different groups of tumor bearing mice under NU-7441 treatment, *n* = 6. **j** Quantitative distribution of tumor infiltrating T lymphocytes in different groups, *n* = 6. Data are presented as the mean ± SD, Student's t-test was used. *P < 0.05, **P < 0.01, ***P < 0.001, ****P < 0.0001

MiRNAs usually bind target 3'UTR regions and degrade target genes. To validate the function of miR-375, We performed data analysis on the previous data of GSE147698 to find differentially expressed mRNAs. A total of 1777 up-regulated genes and 262 down-regulated genes were detected on the microarray overexpressing miR-375 (Fig. 5a). Dramatically, we found a link between miR-375 and the parental gene PRKDC. We hunted the database (starbase.sysu.edu.cn) for miR-375 pairing sequences with the 3'UTR region of the PRKDC gene (Fig. 5b). QRT-PCR experiment found hsa_circ_0136666 is closely related to its parent gene expression. Hsa_circ_0136666 overexpression upregulates the expression of PRKDC, while hsa_circ_0136666 knockdown downregulates the expression of PRKDC (Supplementary 5a-b). Meanwhile, there is a significant negative correlation between miR-375 and PRKDC mRNA levels (Supplementary 5c-d). Complementary paired sequences were confirmed in RIP and dual luciferase reporter experiments (Fig. 5c-d and Supplementary Fig. 5f).

We found that PRKDC mRNA was mainly in the cytoplasm, and we also observed an almost identical distribution with miR-375 (Fig. 5e). By forming RISC complex, miRNA combines with target gene mRNA 3'UTR, resulting in inhibition of mRNA translation. MiR-375 can simultaneously regulate the expression of PRKDC encoded protein DNA-PKcs. It is the catalytic subunit of DNA dependent protein kinase (DNA-PK) and functions as a Ku70/Ku80 heterodimeric protein in DNA double strand break repair and recombination. Total protein expression was measured after transfection of miR-375 mimics and miR-375 inhibitor in MKN-45 and AGS cells, repectively. It was found that miR-375 silenced PRKDC expression, leading to DNA-PK downregulation (Fig. 5f and Supplementary Fig. 5g-j). DNA-PK autophosphorylation is the active state of DNA-PK protein, and its phosphorylation level reflects the activity. We found that hsa_circ_0136666 overexpression accompanied by the upregulation of p-DNAPK, no significance though. DNA-PK is a member of the PI3K kinase superfamily, while downregulation of DNA-PK did not alter Akt/p-Akt. Therefore, we can only infer that promoting DNAPK expression through hsa_circ_0136666 has a relatively small impact on Akt, and hsa_circ_0136666 may not promote cancer development through the Akt/mTOR pathway.

Furthermore, inhibition of DNA-PK activity resulted in excellent tumor suppression. We found that a high concentration of NU-7441 (a DNA-PK selective inhibitor) showed great tumor inhibitory effects (Fig. 5g-h). A high concentration of NU-7441 also resulted in a significant increase in T cell recruitment compared to the control (Fig. 5i), which activated the immune system, thus killing cancer cells effectively (Fig. 5j). We explored that hsa_circ_0136666 functioned as a miR-375 sponge to prevent miRNAs from degrading target genes, thus enabling DNA-PK to exert a pro-oncogenic effect, leading to the occurrence of immune escape.

### Protein kinase DNA-PK drives PD-L1 T20T22 dual-site phosphorylation to mediate protein stabilization

**Fig. 6:**
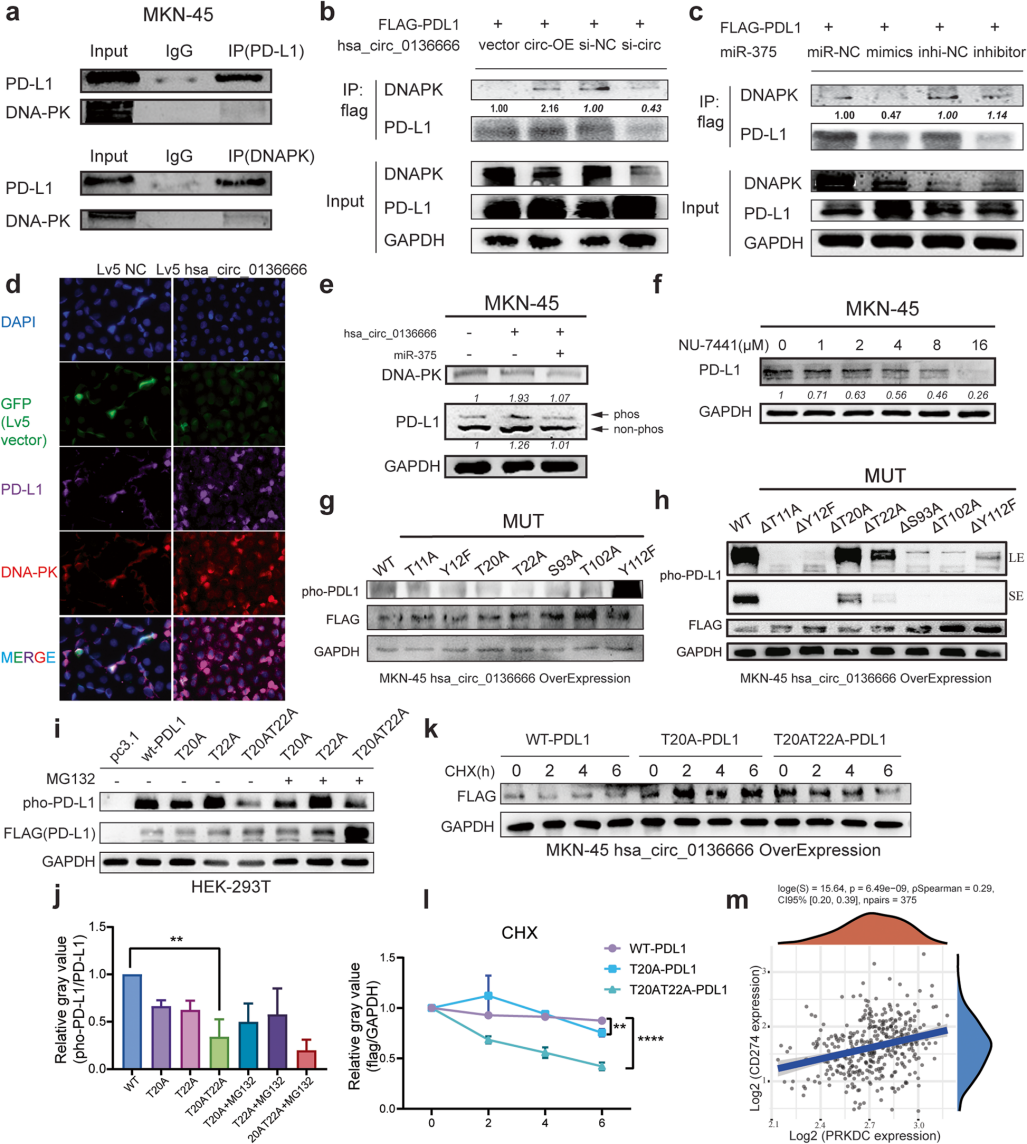
Protein kinase DNA-PK drives PD-L1 T20T22 dual-site phosphorylation to mediate protein stabilization. **a** Endogenous co-immunoprecipitation was used to verify the binding of DNA-PK to PD-L1. **b** Exogenous co-immunoprecipitation was used to verify the binding of DNA-PK and PD-L1 in 293 T cells in the presence of hsa_circ_0136666. **c** Exogenous co-immunoprecipitation was used to verify the binding of DNAPK and PD-L1 in 293 T cells in the presence of miR-375. **d** Immunofluorescence was carried out to confirm the colocalization of DNA-PK and PD-L1. **e** Rescue experiment using phosphorylated acrylamide SDS-PAGE to detect phosphorylated PD-L1. **f** The PRKDC inhibitor NU-7441 decreased PD-L1 protein expression in a concentration dependent manner. **g** Detection of phosphorylated PD-L1 after single mutation of seven phosphosites. **h** Detection of phosphorylated PD-L1 after multiple mutations. **i** Detection of phosphorylated PD-L1 after double phosphorylation site mutation under MG-132 treatment. **j** Quantification of phospho-PD-L1 after mutation of dual phosphorylation sites, *n* = 3. **k** CHX experiment was used to verify the stability of PD-L1 protein. **l** Quantification plot of PD-L1 protein stability, *n* = 3. **m** Clinical data of 375 groups of CD274 and PRKDC genes analyzed by TCGA database. Data are presented as the mean ± SD, Student's t-test was used. **P < 0.01, ****P < 0.0001

We found a strong association between PRKDC and the immune checkpoint PD-L1 through previous experiments. Next, we proposed the hypothesis that protein kinase may directly bind to and phosphorylate PD-L1, mediate PD-L1 protein stability upregulation, and thereby enrich for and promote tumor immune escape. To this end, we first performed endogenous validation in tumor cells. PD-L1 protein can be pulled down using DNA-PK (mAb) and vice versa (Fig. 6a). In HEK-293 T cells transfected with hsa_circ_0136666 or miR-375 mimics, we were able to observe a striking increase in pull-down proteins with upregulation of DNA-PK (Fig. 6b-c). It was also intuitive by immunofluorescence that DNA-PK colocalizes locally with PD-L1 in the cytoplasm (Fig. 6d).

Considering that DNA-PK belongs to the PI3K kinase superfamily, we asked whether PRKDC directly phosphorylates PD-L1. Through rescue experiments, we found that the addition of hsa_circ_0136666 resulted in the upregulation of PD-L1 phosphorylation, and miR-375 could partially eliminate this up-regulation (Fig. 6e). NU-7441 can inhibit DNA-PK kinase activity, and we also found that PD-L1 expression was inhibited by NU-7441 (Fig. 6f).

After verifying that DNA-PK directly binds to PD-L1, we further demonstrated that DNA-PK drives PD-L1 phosphorylation. We resorted to mass spectrometry analysis after DNA-PK antibody pull-down in HEK-293 T cells, and by modification mass spectrometry analysis, we found seven potential PD-L1 phosphorylation sites (Supplementary Fig. 6a). To confirm that there are potential sites of PD-L1 phosphorylation by DNA-PK, we mutated these seven sites and examined the phosphorylated proteins using an isolated gel in the phosbind acrylamide configuration. We have identified two sites (T20, T22) where the phosphorylated protein levels were significantly downregulated (Fig. 6g). In contrast, the levels of phosphorylated proteins were upregulated upon keeping only a single site unmutated (Fig. 6h). Moreover, PD-L1 double phosphorylation site (T20AT22A) mutations downregulated PD-L1 phosphorylation more than single site mutation at MKN-45 hsa_circ_0136666 overexpressing cell (Fig. 6i-j). And PD-L1 was less stable than the wild-type protein and degraded more within 6 h under CHX treatment (Fig. 6k-l). Clinical data also supported the extremely high positive correlation of DNA-PK and PD-L1 in mixed gastric adenocarcinoma tissue samples (Fig. 6m).

In summary, we found direct binding and colocalization of the protein kinase DNA-PK with PD-L1. DNA-PK phosphorylated PD-L1 at the T20T22 double site, making PD-L1 less susceptible to degradation and elevating PD-L1 protein stabilization, with aggregation of PD-L1 to promote immune dysregulation.

### Hsa_circ_0136666 is a novel drug target that siRNA can effectively improve anti-PD-L1 drug efficacy

**Fig. 7:**
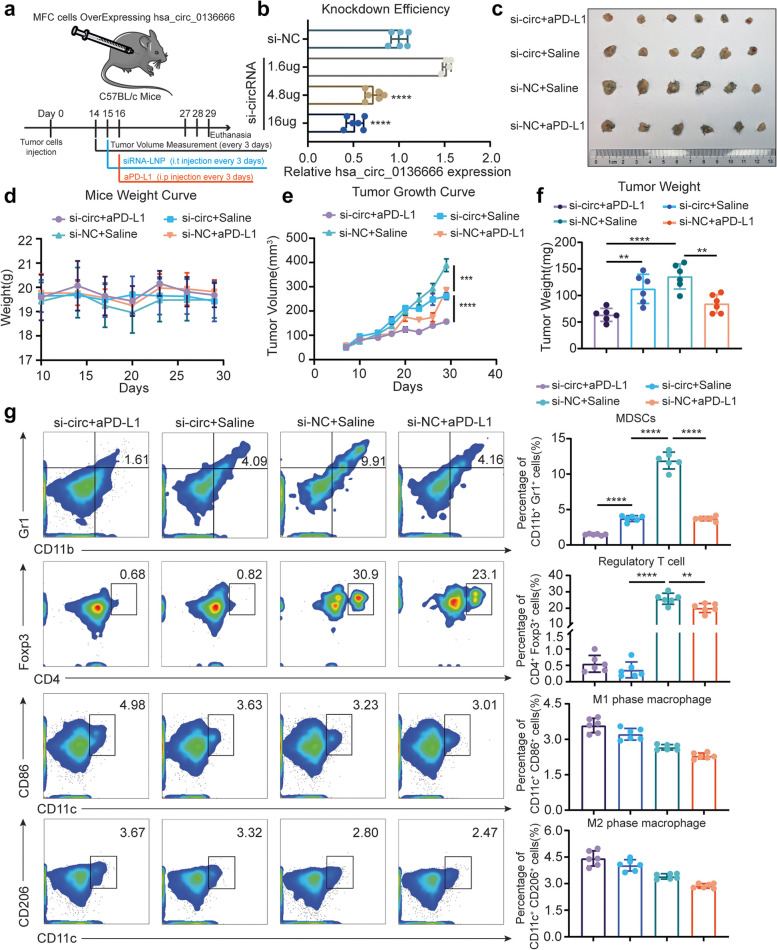
Hsa_circ_0136666 is a novel drug target that siRNA can effectively improve anti-PD-L1 drug efficacy (**a**) Schematic diagram of animal dosage. 14 days after tumor inoculation, mice were injected with LNP-siRNA at a dose of 2 mg/kg intratumorally, and 2 mg/kg mouse PD-L1 inhibitor was injected intraperitoneally the next day. The dosing cycle was three days for five times. **b** QRT-PCR was used to detect the knockdown efficiency of siRNA, *n* = 3. **c** Original photo of tumor in tumor bearing mice under LNP and inhibitor treatment. **d** Body weight curve of tumor bearing mice, *n* = 6. **e** Tumor growth curve of tumor bearing mice, *n* = 6. **f** Comparison of tumor weight in mice, *n* = 6. **g** Distribution difference and quantitative analysis of immunosuppressive cells in tumor area of tumor bearing mice among groups, *n* = 6. **P < 0.01, ***P < 0.001, ****P < 0.0001

To verify whether hsa_circ_0136666 can serve as an effective drug target, we established a subcutaneous model in C57BL/c mice (Fig. 7a). Briefly, MFC cells overexpressing hsa_circ_0136666 tumor-bearing mice were randomly divided into four groups and intratumorally injected with a nanoliposome encapsulated siRNA concentrate (equivalent of 40 µg siRNA per mouse, 100 µl stock solution per 40 µg siRNA). The knockdown efficiency of si-circRNA can reach 50% at the cellular level (Fig. 7b). Meanwhile, we also administered 2 mg/kg (anti-mouse PD-L1 mAb) by intraperitoneal injection, and both drugs were injected every three days. We found that the tumor growth was significantly inhibited in the LNP-siRNA group compared with the saline & LNP-siNC group, and the tumor volume of the LNP-siRNA & aPD-L1 group was smaller than that of the aPD-L1 monotherapy group. The tumor size and weight of the LNP-siRNA & aPD-L1 group were the smallest among the four groups, and the inhibition rate was approximately 50–60% compared with the control group, calculated by the size and weight of the tumor (Fig. 7c-f). We know that there was no significant change in the body weight of mice in the four groups, indicating that the two drugs had no obvious toxicity (Fig. 7d). We considered that the siRNA can be driven to act on the tumor area most effectively by intratumoral injection, and the degradation of siRNA by nucleases in the blood can be avoided by the nanoliposome encapsulated drug delivery system.

As we first envisioned, siRNA partially inhibited tumor growth, but this inhibition rate was not significant compared to mAb treatment. However, we found that LNP-siRNA was helpful for the treatment of tumor microenvironment, and this effect was reflected in the proportion of immunosuppressive cells. MDSCs were significantly suppressed in tumor regions by LNP-siRNA treatment, and the number of MDSCs in the LNP-siRNA & aPD-L1 group was the smallest among the four groups. Similarly, the number of tumor-infiltrating regulatory T cells was the less in all LNP-siRNA treated groups and greater in the group without LNP-siRNA treatment. However, data related to tumor-associated macrophages did not show significant changes (Fig. 7g).

We wanted to study effector CD8^+^ T cells in the tumor area, including granzyme B released by T cells. The results of immunofluorescence staining for tumor areas showed that the LNP-siRNA & aPD-L1 group had the widest distribution of CD8^+^ T cells, accompanied by the highest expression of granzyme B and the lowest expression of PD-L1. The expression of PD-L1 in the LNP-siRNA group and the aPD-L1 group were all downregulated compared with the negative control group (Supplementary Fig. 7).

Taken together, these results suggested that LNP-siRNA can effectively improve the anti-tumor effect of anti-PD-L1 drugs, and significantly hinder the recruitment of immunosuppressive cells. LNP-siRNA possessed no evident side effects, and can be a safe agent for further in vivo applications.

## Discussion

Over the past decade, circRNAs have emerged as a large class of mainly non coding RNA molecules that play critical roles in cancer initiation and progression through diverse mechanisms of action. Later accumulating evidence showed that circRNAs also encode entirely new short peptides regulating cancer. The key physiological functions of circRNAs include miRNA sponges, transcriptional regulators, and association with RNA binding proteins to exert important biological functions by modulating protein functions or encoding cryptic peptides. Regarding hsa_circ_0136666 encodes a recessive peptide, we did not investigate. To best of our knowledge, hsa_circ_0136666 does not have a ribosome binding site, which is called the IRES region. There is no ATG promoter on the loop structure on hsa_circ_0136666. The above reasons led us to the conclusion that hsa_circ_0136666 does not have the ability to encode a completely new micro peptide.

**Fig. 8:**
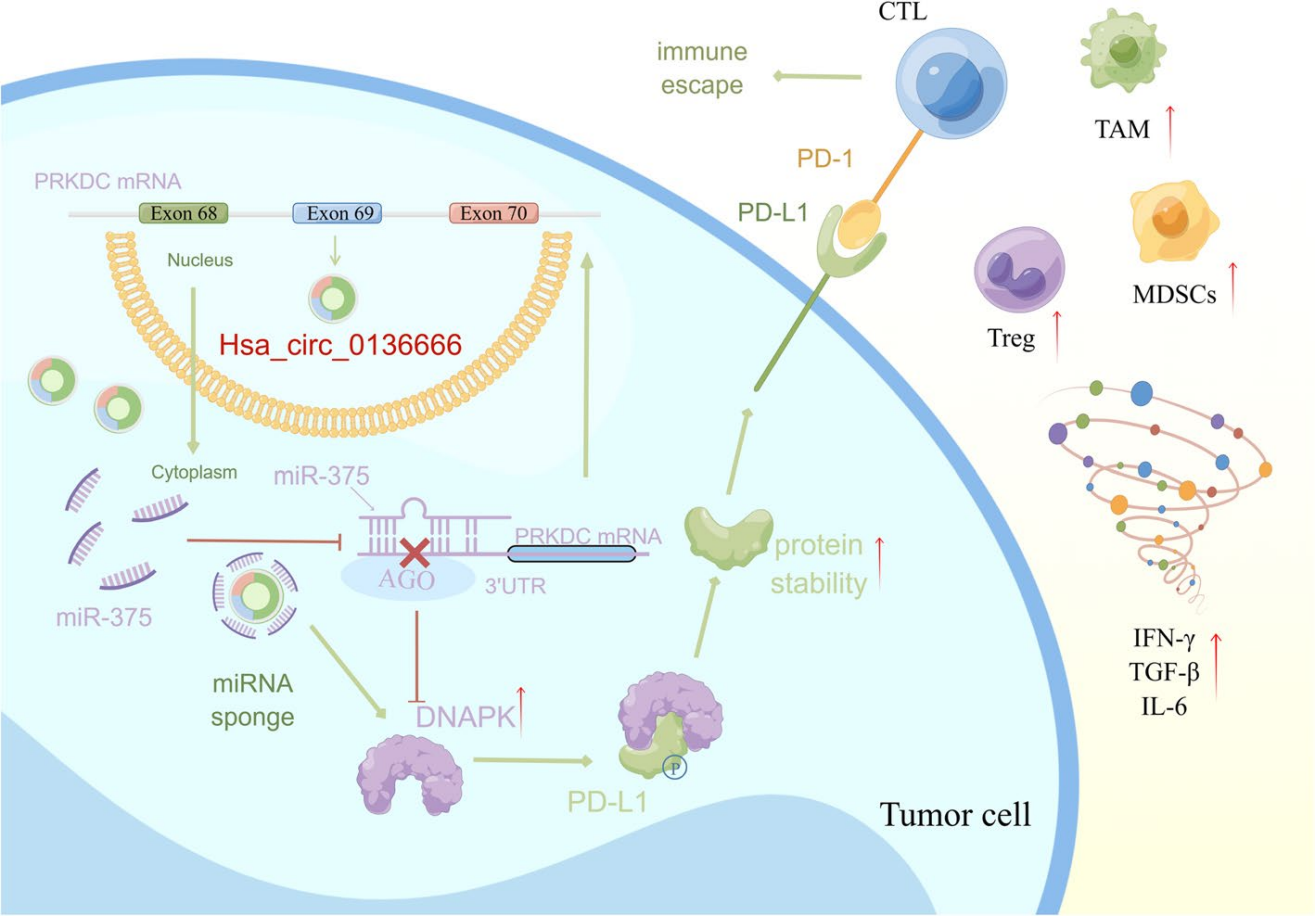
The signal pathway diagram of hsa_circ_0136666 promoting gastric cancer progression. Specifically, hsa_circ_0136666/miR-375/PRKDC upregulates DNA-PK protein expression, driving PD-L1 phosphorylation. The increased stability of PD-L1 results in abnormal aggregation on the surface of the cell membrane, thus leading to immune escape

In this article, we investigate the miRNA sponge function of hsa_circ_0136666. This is a hot and important point of research in recent years. CircRNAs affect on tumor growth through sponge function have been seen in a large number of reports. However, most studies focus on tumor cell proliferation, invasion, and migration, and few studies have focused on the immunological function of circRNAs. Thus, the main finding of our study is hsa_circ_0136666 functions as a miR-375 sponge to repress the function of miR-375 gene silence and to prevent PRKDC gene silencing. The phenomenon of immune escape occurs as a result of increased DNA-PK protein translation leading to PD-L1 protein interactions, phosphorylation of PD-L1, and aberrant clustering to the cell membrane surface (Fig. 8).

Small nucleic acid drugs are also known as RNA interference(RNAi) technology drugs. Small nucleic acid drugs are a completely new class of drugs completely different from small molecule drugs, antibody drugs, whose composition is nucleotide sequence, drug mechanism is to act on mRNA and inhibit the expression of target proteins through gene silencing, so as to achieve the purpose of treating diseases [29]. The range of small nucleic acid drugs covers siRNA, miRNA, and antisense nucleic acids, among others. RNAi technology research was awarded the Nobel Prize in physiology or medicine in 2006 for major discoveries and technological innovation in the history of human science. In principle, RNAi could be used to treat any disease associated with elevated expression of identified genes, including the treatment of viral diseases, cancer, and inflammatory diseases [30–33]. Therefore, research on the function and molecular mechanism of small nucleic acid drugs in cancer is an important research direction to develop biotechnological drugs for the treatment and prevention of gastric cancer, which can provide new ideas for drug target discovery, clinical diagnosis, and treatment. Lipid nanoparticles provide a promising solution to the difficulty of effective delivery of siRNA, with the potential to enhance the efficacy and biocompatibility of nucleic acid drugs.

The efficiency of gene silencing by naked siRNA in vivo is extremely low because naked siRNA molecules are rapidly degraded by nucleases in the blood and rapidly undergo renal clearance in vivo. In addition, the bulkiness and negative charge of siRNA hinder its penetration through the cell membrane, hindering its intracellular accumulation. Therefore, efficient delivery is critical to bringing siRNA to target cells and tissues. Liposomes are made from the inclusion of phospholipids as membrane materials, which are the basic materials to form the bilayer of liposomes and have good biocompatibility. Liposomes are a kind of drug-loading means used in medicine and pharmacy, mainly used to enhance the transdermal absorption ability of drugs.

Our study also has several weaknesses. In the discovery of circRNAs, we chose to study hsa_circ_0136666 as its parental gene is also part of a regulatory loop. But there are other differentially expressed circRNAs, which we did not investigate. Second, we did not find hsa_circ_0136666 other functions including those as RNA-binding proteins. We can't account for hsa_circ_0136666 modulations of tumor proliferation and immune escape by miR-375 sponge function alone. Third, in terms of applications, the tumor inhibition effect of siRNA-LNP alone is not obvious, there is a partial tumor inhibition effect with the combination of PD-L1 inhibitors and siRNA-LNP, but both targets are PD-L1, so the functionally limited drug efficacy is difficult to break through, so it also needs to be combined with other drugs or to humanize mouse experiments to further illustrate the drug efficacy of siRNA.

## Methods & materials

### Human tissue specimens

21 cases of gastric cancer recurrence and metastasis: 7 cases of primary tumor/adjacent-tumor/metastasis, 5 cases of primary tumor/adjacent-tumor, 5 cases of primary tumor/metastasis, and 4 cases of metastasis. There were 5 cases of normal human gastric mucosa. A total of 50 cases of clinicopathological tissue. The study protocol was approved by the Shandong University Research Ethics Committee and Taizhou Hospital Ethics Committee/Shanghai Outdo Biotech Company Ethics Committee. Detailed information of 21 Gastric cancer cases of tissue microarray was in Supplementary Table 1.

### Cell culture and transfection

GES-1, MKN-45 and MFC were cultured in RPMI-1640 medium, HEK-293 T was in Dulbecco's Modified Eagle medium (DMEM; keyGEN, China). All cells were purchased from Cell Bank of Chinese Academy of Sciences and cultured with medium supplemented with 10% fetal bovine serum (FBS; LONSERA, Shanghai, China) at 37℃ in 5% CO2 incubator. AGS was purchased from Cell Bank of Chinese Academy of Sciences and cultured in AGS cell specific medium (Procell, CM-0022). All cells used for experiments were in logarithmic growth phase, within 20 passages. Cells were transfected with jetPRIME® (Polyplus-transfection, Inc., New York, USA) for siRNAs or miRNA mimics and plasmids.

### Actinomycin D assay

Cells were exposed to DMSO or 2 µg/mL actinomycin D (APExBIO, USA) to block transcription for 2, 4, 8, and 12 h. Then the expression of hsa_circ_0136666 and its linear transcript PRKDC mRNA were detected using QRT-PCR.

### Ribonuclease R (RNase R) digestion

Total RNA (1 µg) was incubated for 10 min at 37 °C with or without 1 U/µg RNase R (Geenseed, Guangzhou, China) according to manufacturer’s instructions and then reverse-transcribed to cDNA. Then, the expression of hsa_circ_0136666 and its linear transcript PRKDC mRNA were detected using QRT-PCR.

### RNA fluorescence in situ hybridization (FISH)

Cy3-labeled oligonucleotide probe for miR-375 and FITC-labeled oligonucleotide probe for hsa_circ_0136666 and PRKDC mRNA were applied for RNA FISH. MiR-375 and PRKDC mRNA were designed and synthesized by GenePharma (Shanghai, China), hsa_circ_0136666 probe was by Geneseed (Guangzhou, China). The hybridization was performed in MKN-45 cells, including hsa_circ_0136666 & miR-375 and miR-375 & PRKDC mRNA. Cell slides fixed with 4% paraformaldehyde were treated with several buffers in RNA-FISH Kit (GenePharma). After denaturing at 73 °C for 5 min, probe mixture was hybridized in cell slide overnight in darkness at 37 °C. Scans were acquired using a laser confocal scanner, and images were viewed and exported with CaseViewer.

### RNA isolation and quantitative reverse transcription-PCR (QRT-PCR)

Total RNAs of cells and tissue samples were isolated using TRIzol reagent (TransGen Biotech, BeiJing, China). In order to quantify mRNA and circRNA, RNAs were reversely transcribed into cDNA using HiScript III RT SuperMix (R323-01, Vazyme), quantitative reverse transcription-PCR was conducted using ChamQ Universal SYBR qPCR Master Mix (Q711-02, Vazyme). The reverse transcription of miRNA use M-MLV (H-) Reverse Transcriptase (R021-01, Vazyme). Relative circRNA, mRNA or miRNA expression was normalized to GAPDH or U6 snRNA levels, using the 2^−ΔΔCt^ method. The sequence for each primer was listed in Supplementary Table 2. The average threshold cycle for each gene was determined from at least three independent experiments.

### CD8^+^ T Cell coculture assay

CD8^+^ T cells derived from spleen were isolated using the MojoSort™ Mouse CD8^+^ T Cell Isolation Kit (catalog 480,007, BioLegend). The isolated CD8^+^ T cells were activated by Ultra-LEAF™ Purified anti-mouse CD3ε/CD28 (catalog 100,339/102115, BioLegend) for 3 days according to the manufacturer’s protocol. The experiments were performed in RPMI-1640 with IL-2 (10 ng/mL). Lentiviral transfected cancer cells were allowed to adhere to the plate overnight and then incubated for 48 h with activated CD8^+^ T cell.The ratio between cancer cells and CD8^+^ TILs ranged from 1:1 to 1:20. T cells and cell debris were removed by PBS wash, and living cancer cells were then quantified by a spectrometer at OD 595 nm followed by crystal violet staining.

### Luciferase reporter assay

Hsa_circ_0136666 or mut-hsa_circ_0136666, miR-375 mimics or miR-NC, PRKDC 3'UTR or mut-PRKDC 3'UTR, pMIR-Report were co-transfected in HEK-293 T cells. The relative luciferase activity was measured with the Duo-Lite Luciferase Assay System (Vazyme, China) according to the manufacturer’s instructions after 48 h. Fluorescence values were detected by multifunctional microplate reader i3x at 560 nm (Firefly luciferase) and 480 nm (Ranilla luciferase).

### RNA-binding protein immunoprecipitation (RIP)

RIP assay was performed occupying the Protein A/G Agarose Resin (Bimake, USA) following the manufacturer’s protocol. Briefly, hsa_circ_0136666 or mut-hsa_circ_0136666, miR-375 mimics or miR-NC, PRKDC 3'UTR or mut-PRKDC 3'UTR were co-transfected in MKN-45 cells. Cells were harvested after 48 h post transfection and lysed by NP-40 lysis buffer (Beyotime, China). After centrifugation, the supernatant was coincubated with human AGO2 antibody. Protein-A/G agarose beads were binding to the AGO2 antibody for 3 h. RNA was then extracted from the immune complex and the immunoprecipitated RNA was used for QRT-PCR analysis.

### Cell viability assay (Cell Counting Kit-8)

Cell viability was assessed using CCK-8 (Cell Counting Kit-8) assay (Target Mol, USA). MKN-45 and AGS were transfected with hsa_circ_0136666 plasmid or siRNA using jetPRIME. Cells were seeded in a 96-well plate at a density of 2000 cells/well in 100 μL of culture medium in a 5% CO_2_ incubator at 37 °C for 24 h. After incubating for 24 h, 10 μL CCK-8 was added to each well for 1 h incubation. The absorbance was measured at 450 nm with a microplate reader.

### Western blotting and co-immunoprecipitation

Total protein was extracted using RIPA buffer (EpiZyme, Shanghai, China) and quantified with the BCA Protein Assay Kit (YEASEN, China). The PAGE Gel Fast Preparation Kit was used to prepare SDS-PAGE (EpiZyme, Shanghai, China). The protein was incubated in primary antibodies overnight at 4 °C. The antibodies used in this assay are listed in Supplementary Table 3.

### Enzyme-linked immunosorbent assay(ELISA)

Mice tumor weight was accurately weighed, saline was added in the ratio of weight (mg): Volume (μL) = 1:9, grinder homogenized under ice water bath conditions. Experiments were performed according to the mouse ELISA Kit for IL-6, TGF-β1 and IFN-γ (Multiscience) instructions. The standard curve was derived using a linear fit.

### Use of siRNA and preparation of LNP

For siRNA (Biomics) encapsulation into LNP, lipid components were dissolved in ethanol at molar ratios of 50:10:38.5:1.5 (ionizable lipid: DSPC: cholesterol: PEG-lipid). The lipid mixture was combined with a 10-mM citrate buffer (pH 4.0) containing siRNA at a volume ratio of 1:3 (lipids: siRNA) using a NanoAssembler system (Suzhou Wenhao Microfluidic Technology Co., Ltd., China). Formulations were centrifuged and concentrated using Amicon Ultra-15 ultrafiltration unit. SiNC and siRNA were synthesized by biomics biotech, hsa_circ_0136666 siRNAs and shRNAs sequences are listed in Supplementary Table 4.

### Animal model construction and dosing regimen

In general, C57BL/c or BALB/c Nude mice were inoculated subcutaneously with tumor cells, and tumor volume was measured and administered starting from the time tumors grew to 100 mm^3^. Anti-mouse PD-L1 mAb was administered by intraperitoneal injection at a dose of 2 mg/kg every three days. LNP-siRNA was intratumorally injected into the tumor area with an administration dose of 40 μg siRNA (siNC) per mouse. The dosing volume did not exceed 100ul. The dosing frequency was once every three days (Time staggered with aPD-L1). The CDX model referred to the general subcutaneous tumor grafting procedure.

### Flow cytometric analysis in mouse tumors

Mice were sacrificed by the spinal dislocation method, the tumors were stripped intact using surgical instruments sterilized with 75% ethanol, and the tumors were cut into small pieces using sterile ophthalmic surgical scissors and digested using 0.25% trypsin for 10–20 min. Single-cell suspensions were prepared by filtering through a 300-mesh nylon mesh two or three times, and the cell concentration was adjusted to reach about 5 × 10^5^/ml. Adjusted single-cell suspensions were stained with antibodies for at least 20 min according to standard protocols. Staining protocols are listed in Supplementary Table 5. Stained cells were analyzed using a BD FACS Celesta (BD Biosciences). Data were processed using the FlowJo software program.

## Statistical analysis

All experiments were carried out a minimum of three times unless otherwise stated. Quantitative data are represented as the means ± SD, and compared statistically by unpaired Student’s t test, using SigmaPlot (12.0). Statistical significance was indicated as follows: *P ≤ 0.05, **P ≤ 0.01, ***P ≤ 0.001, ****P ≤ 0.0001. Graphs were generated using GraphPad Prism (8.3.0) or FlowJO (V10).

### Supplementary Information

**Additional file 1: Supplementary Table 1.** Detailed information of 21 Gastric cancer cases of tissue microarray. **Supplementary Table 2.** The primers were used for quantitative reverse transcription-PCR (QRT-PCR). **Supplementary Table 3.** The antibodies were used for Western blot and co-immunoprecipitation. **Supplementary Table 4.** SiRNAs & shRNA were used for circRNA knockdown. **Supplementary Table 5.** All the antibodies used for flow cytometry were purchased from BioLegend. **Supplementary Figure 1.** (a) TCGA database analysis of patient survival Under high/low expression of miR-375. (b) TCGA database analysis of patient survival Under high/low expression of PRKDC. **Supplementary Figure 2.** GO analysis and KEGG analysis of gene chips overexpressed in miR-375. **Supplementary Figure 3. **(a) Original photo of tumor in tumor bearing mice overexpressing or knocking down hsa_circ_0136666. (b) Comparison of tumor weight in tumor bearing mice, *n=*6. (c) Differential expression of IFN-γ in tumor regions of tumor bearing mice, *n=*4. (d) Differential expression of IL-6 in tumor regions of tumor bearing mice, *n=*4. (e) Differential expression of TGF-β in tumor regions of tumor bearing mice, *n=*4. (f) IHC staining of tumor sections in tumor bearing mice, five visual field ranges were randomly selected for quantification. Data are presented as the mean ± SD, Student's t-test was used. *P<0.05, **P<0.01, ***P<0.001. **Supplementary Figure 4.** (a) QRT-PCR was used to detect mRNA levels of immune checkpoint in MKN-45 cell overexpressing hsa_circ_0136666. (b) QRT-PCR was used to detect mRNA levels of immune checkpoint in MKN-45 cell with knockdown of hsa_circ_0136666. (c) QRT-PCR was used to detect mRNA levels of immune checkpoint in AGS cell overexpressing hsa_circ_0136666. (d) QRT-PCR was used to detect mRNA levels of immune checkpoint in AGS cell with knockdown of hsa_circ_0136666. Data are presented as the mean ± SD, *n=*3, Student's t-test was used, *P<0.05, **P<0.01, ***P<0.001. (e-h) Quantification diagram of Western blot in Fig 2l-m. *P<0.05, ***P<0.001. **Supplementary Figure 5.** (a) QRT-PCR detection was used to detect a positive correlation between hsa_circ_0136666 and PRKDC expression in AGS cell. (b) QRT-PCR detection was used to detect a positive correlation between hsa_circ_0136666 and PRKDC expression in MKN-45 cell. (c) QRT-PCR detection was used to detect a negative correlation between miR-375 and PRKDC expression in AGS cell. (d) QRT-PCR detection was used to detect a negative correlation between miR-375 and PRKDC expression in MKN-45 cell. (e) RIP experiment was used to verify that hsa_circ_0136666 exerts sponge function. (f) RIP experiment was used to verify the combination of miR-375 and PRKDC. Data are presented as the mean ± SD, *n=*3, Student's t-test was used, *P<0.05, **P<0.01, ***P<0.001,****P<0.0001. (g-j) Quantification diagram of Western blot in Fig 5f, *P<0.05, **P<0.01, ***P<0.001. **Supplementary Figure 6.** (a) Biomass spectrometry has been implemented to detect 7 possible phosphorylation sites. (b) T20T22 phosphorylated PD-L1 mass spectrometry chart. (c) S93T102 phosphorylated PD-L1 mass spectrometry chart. **Supplementary Figure 7.** Immunofluorescence staining of tumor slices from tumor bearing mice was performed to detect immune indicators and checkpoint proteins.

## Data Availability

All data generated or analysed during this study are included in this published article [and its supplementary information files].

## Abbreviations

aPD-L1: PD-L1 antibody
CircRNA: Circle RNA
DNAPK: DNA-dependent protein kinase
GAPDH: Glyceraldehyde-3-phosphate dehydrogenase
IFN-γ: Interferon-γ
IL-6: Interleukin-6
LNP: Lipid nanoparticles
MDSCs: Myeloid-derived suppressor cells
MiRNA: Micro RNA
MUT: Mutant type
NC: Negative control
PBS: Phosphate buffered saline
PD-L1: Programmed cell death 1 ligand 1
PRKDC: Protein kinase, DNA-activated, Catalytic subunit
SiRNA: Small interfering RNA
TAM: Tumor associated macrophages
TEST: Tris buffer saline-Tween 20
TGF-β: Transforming growth factor-β
Treg: Regulatory T cell
UTR: Untranslated region
WT: Wild type
