# Supplementary material for: Hsa_circ_0136666 stimulates gastric cancer progression and tumor immune escape by regulating the miR-375/PRKDC Axis and PD-L1 phosphorylation
Source: Mol Cancer. 2023 Dec 13;22:205. doi: 10.1186/s12943-023-01883-y (PMC10718020; doi:10.1186/s12943-023-01883-y)
Supplement: Supplementary file 1 — Additional file 1: Supplementary Table 1. Detailed information of 21 Gastric cancer cases of tissue microarray. Supplementary Table 2. The primers were used for quantitative reverse transcription-PCR (QRT-PCR). Supplementary Table 3. The antibodies were used for Western blot and co-immunoprecipitation. Supplementary Table 4. SiRNAs & shRNA were used for circRNA knockdown. Supplementary Table 5. All the antibodies used for flow cytometry were purchased from BioLegend. Supplementary Figure 1. (a) TCGA database analysis of patient survival Under high/low expression of miR-375. (b) TCGA database analysis of patient survival Under high/low expression of PRKDC. Supplementary Figure 2. GO analysis and KEGG analysis of gene chips overexpressed in miR-375. Supplementary Figure 3. (a) Original photo of tumor in tumor bearing mice overexpressing or knocking down hsa_circ_0136666. (b) Comparison of tumor weight in tumor bearing mice, n=6. (c) Differential expression of IFN-γ in tumor regions of tumor bearing mice, n=4. (d) Differential expression of IL-6 in tumor regions of tumor bearing mice, n=4. (e) Differential expression of TGF-β in tumor regions of tumor bearing mice, n=4. (f) IHC staining of tumor sections in tumor bearing mice, five visual field ranges were randomly selected for quantification. Data are presented as the mean ± SD, Student's t-test was used. *P<0.05, **P<0.01, ***P<0.001. Supplementary Figure 4. (a) QRT-PCR was used to detect mRNA levels of immune checkpoint in MKN-45 cell overexpressing hsa_circ_0136666. (b) QRT-PCR was used to detect mRNA levels of immune checkpoint in MKN-45 cell with knockdown of hsa_circ_0136666. (c) QRT-PCR was used to detect mRNA levels of immune checkpoint in AGS cell overexpressing hsa_circ_0136666. (d) QRT-PCR was used to detect mRNA levels of immune checkpoint in AGS cell with knockdown of hsa_circ_0136666. Data are presented as the mean ± SD, n=3, Student's t-test was used, *P<0.05, **P<0.01, ***P<0.001. (e-h) Quantification [file 12943_2023_1883_MOESM1_ESM.docx]

**Supplementary Table 1**

**Detailed information of 21 Gastric cancer cases of tissue microarray.**

| Patient number | Gender | Age | Organization type | T | N | M | AJCC clinical stage |
| --- | --- | --- | --- | --- | --- | --- | --- |
| 1 | Female | 62 | Primary/Adjacent tumor |  | N0 | M0 | —— |
|  |  | 63 | Metastases |  |  | M1 |  |
| 2 | Female | 61 | Primary/Adjacent tumor |  | —— | M1 |  |
|  |  | 63 | Metastases | T3 | N2 | M1 | 4 |
| 3 | Male | 59 | Primary/Adjacent tumor |  |  | M0 | —— |
|  |  | 60 | Metastases |  | —— | M1 |  |
| 4 | Male | 59 | Primary/Adjacent tumor |  | N1 | M0 | —— |
|  |  | 60 | Metastases |  |  | M1 |  |
| 5 | Female | 50 | Primary/Adjacent tumor |  | N3a | M0 | —— |
|  |  | 51 | Metastases |  |  | M1 |  |
| 6 | Female | 36 | Primary/Adjacent tumor |  | N3b | M0 | —— |
|  |  | 37 | Metastases |  |  | M1 |  |
| 7 | Female | 45 | Primary/Adjacent tumor |  | N3a | M0 | —— |
|  |  | 47 | Metastases |  |  | M1 |  |
| 8 | Male | 55 | Primary/Adjacent tumor | T2 | N0 | M0 | 1B |
| 9 | Male | 53 | Primary/Adjacent tumor | T4a | N3b | M0 | 3C |
| 10 | Female | 41 | Primary/Adjacent tumor | T3 | N3a | M0 | 3B |
| 11 | Female | 56 | Primary/Adjacent tumor | T3 | N2 | M0 | 3A |
| 12 | Male | 50 | Primary/Adjacent tumor | T3 | N3a | M0 | 3B |
| 13 | Female | 59 | Primary |  | N3a | M0 | —— |
|  |  | 59 | Metastases |  |  | M1 |  |
| 14 | Female | 40 | Primary |  |  | M0 | —— |
|  |  | 43 | Metastases |  | —— | M1 |  |
| 15 | Female | 52 | Primary |  |  | M0 | —— |
|  |  | 53 | Metastases |  |  | M1 |  |
| 16 | Male | 63 | Primary |  | N3b | M0 | —— |
|  |  | 64 | Metastases |  |  | M1 |  |
| 17 | Female | 41 | Primary |  |  | M0 | —— |
|  |  | 41 | Metastases |  |  | M1 |  |
| 18 | Female | 44 | Metastases |  |  | M1 |  |
| 19 | Female | 36 | Metastases |  |  | M1 |  |
| 20 | Female | 45 | Metastases |  |  | M1 |  |
| 21 | Male | 54 | Metastases |  |  | M1 |  |

The tissue chip used in this project consisted of 21 cases of gastric cancer recurrence and metastasis. These cases included 7 cases of primary focus/adjacent tumor/metastatic focus, 5 cases of primary focus/adjacent tumor, 5 cases of primary focus/metastasis focus, and 4 cases of metastasis focus. Additionally, there were 5 cases of normal human gastric mucosa.

**Supplementary Table 2**

**The primers were used for quantitative reverse transcription-PCR (qRT-PCR).**

| Name | Primer | Sequence |
| --- | --- | --- |
| *GAPDH* | Forward | GCACCGTCAAGGCTGAGAAC |
|  | Reverse | TGGTGAAGACGCCAGTGGA |
| *Hsa_circ_0136666*  Divergent primer | Forward | CAGAGACGATTGGCTGGTGA |
|  | Reverse | TGATAAATTGCCCAACAAAGAGACT |
| *Hsa_circ_0136666*  Convergent primer | Forward | CAGTGACAGGATGGAAGT |
|  | Reverse | TTTTGACTCTTTATGCAGCTCCTTC |
| *EIF4A3* | Forward | CGCGGACTCTGACATATGGCGACCACGGCCACGATG |
|  | Reverse | TCCCGCAGGCCCATGGTGTCG |
| *PRKDC* | Forward | CTGTGCAACTTCACTAAGTCCA |
|  | Reverse | CAATCTGAGGACGAATTGCCT |
| *PD-L1* | Forward | TGGCATTTGCTGAACGCATTT |
|  | Reverse | TGCAGCCAGGTCTAATTGTTTT |
| *CTLA-4* | Forward | CATGATGGGGAATGAGTTGACC |
|  | Reverse | TCAGTCCTTGGATAGTGAGGTTC |
| *HAVCR2* | Forward | AGACAGTGGGATCTACTGCTG |
|  | Reverse | CCTGGTGGTAAGCATCCTTGG |
| *LAG3* | Forward | GCCTCCGACTGGGTCATTTT |
|  | Reverse | CTTTCCGCTAAGTGGTGATGG |
| *18S rRNA* | Forward | GGACATCTAAGGGCATCACA |
|  | Reverse | ACACGGACAGGATTGACAGA |

**Supplementary Table 3**

**The antibodies were used for Western blot and co-immunoprecipitation.**

| Antibody | Company / Category number |
| --- | --- |
| DNA-PK, Rabbit Monoclonal | Affinity Biosciences, Cat# AF5340 |
| DNA-PK, Mouse Monoclonal | Santa Cruz Biotechnology, Cat# sc-5282 |
| Phospho‐DNA‐PKcs (Ser2056) | Abcam, Cat# ab124918 |
| PD-L1, Rabbit Monoclonal | Proteintech, Cat# 28076-1-AP |
| PD-L1, Mouse Monoclonal | Sino Biological, Cat# 10084-MM33 |
| GAPDH | Proteintech, Cat# 60004-1-Ig |
| TIM3 | Proteintech, Cat# 60355-1-Ig |
| LAG3 | Proteintech, Cat# 16616-1-AP |
| Akt1/2/3 | Abmart, Cat# T55561 |
| mTOR | Abmart, Cat# T55306 |
| Phospho-Akt (Ser473) | Proteintech, Cat# 66444-1-Ig |
| DYKDDDDK tag | Proteintech, Cat# 66008-4-Ig |

**Supplementary Table 4**

| Name | strand | Sequence(5’-3’) |
| --- | --- | --- |
| hsa_circ_0136666-si-1 | sense strand | ACAGUCUCUUUGUUGGGCAAUdTdT |
|  | antisense strand | AUUGCCCAACAAAGAGACUGUdTdT |
| hsa_circ_0136666-si-2 | sense strand | AGUCUCUUUGUUGGGCAAUUUdTdT |
|  | antisense strand | AAAUUGCCCAACAAAGAGACUdTdT |
| hsa_circ_0136666-si-3 | sense strand | GUUGGGCAAUUUAUCAUCUCAdTdT |
|  | antisense strand | UGAGAUGAUAAAUUGCCCAACdTdT |
| Sh- hsa_circ_0136666 | shRNA | GTTGGGCAATTTATCATCTCA |

**Supplementary Table 5**

**All the antibodies used for flow cytometry were purchased from BioLegend.**

| Antibody | Category number |
| --- | --- |
| PE/Cyanine7 anti-mouse CD3 | 100219 |
| PE anti-mouse CD8a | 162303 |
| APC anti-mouse CD4 | 100515 |
| [APC/Cyanine7 anti-mouse CD11c](https://bioec.cn/product/5f601cdaafef2b4993dc8d66) | 117323 |
| [APC anti-mouse CD206 (MMR)](https://bioec.cn/product/5f601ce7afef2b4993dced6b) | 141707 |
| [PE anti-mouse CD86](https://bioec.cn/product/5f601d06afef2b4993ddfdc5) | 159203 |
| [FITC anti-mouse CD4](https://bioec.cn/product/5f601cd7afef2b4993dc7ba7) | 100405 |
| [PE anti-mouse FOXP3](https://bioec.cn/product/5f601cdbafef2b4993dc937c) | 126403 |
| [APC anti-mouse/human CD11b](https://bioec.cn/product/5f601cd7afef2b4993dc7d79) | 101211 |
| [PE/Cyanine5 anti-mouse Ly-6G/Ly-6C (Gr-1)](https://bioec.cn/product/5f601cd9afef2b4993dc8750) | 108409 |
| 7-AAD Viability Staining Solution | 420403 |

Supplementary Figure 1


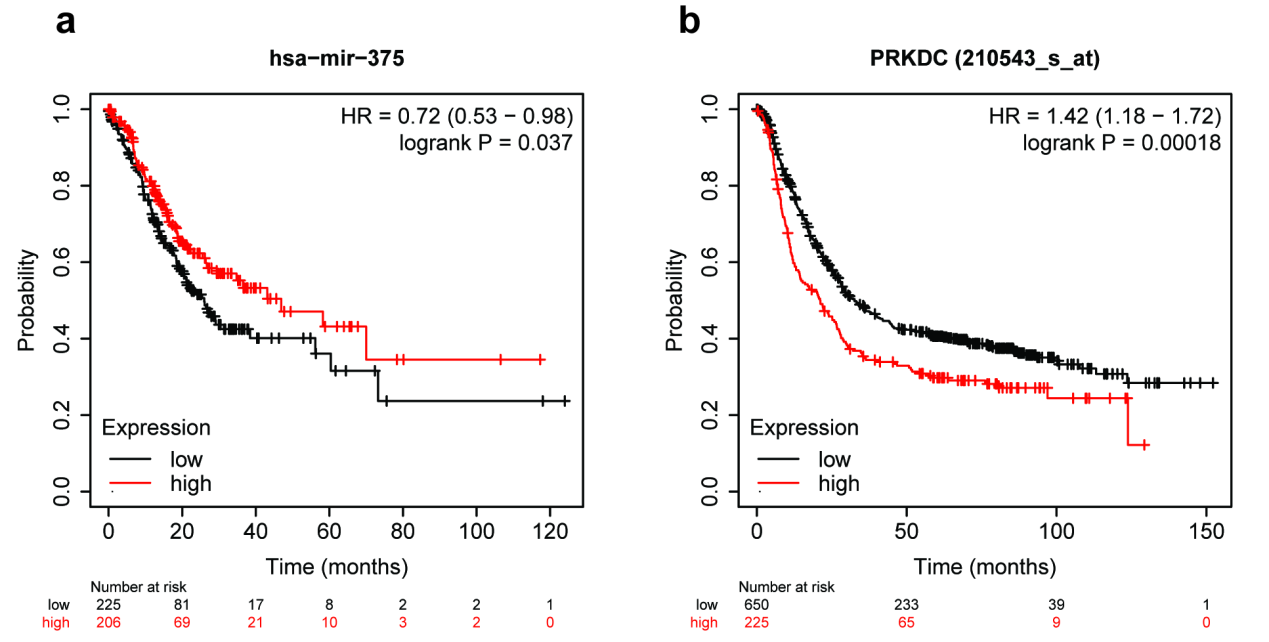


(a) TCGA database analysis of patient survival Under high/low expression of miR-375. (b) TCGA database analysis of patient survival Under high/low expression of PRKDC.

Supplementary Figure 2


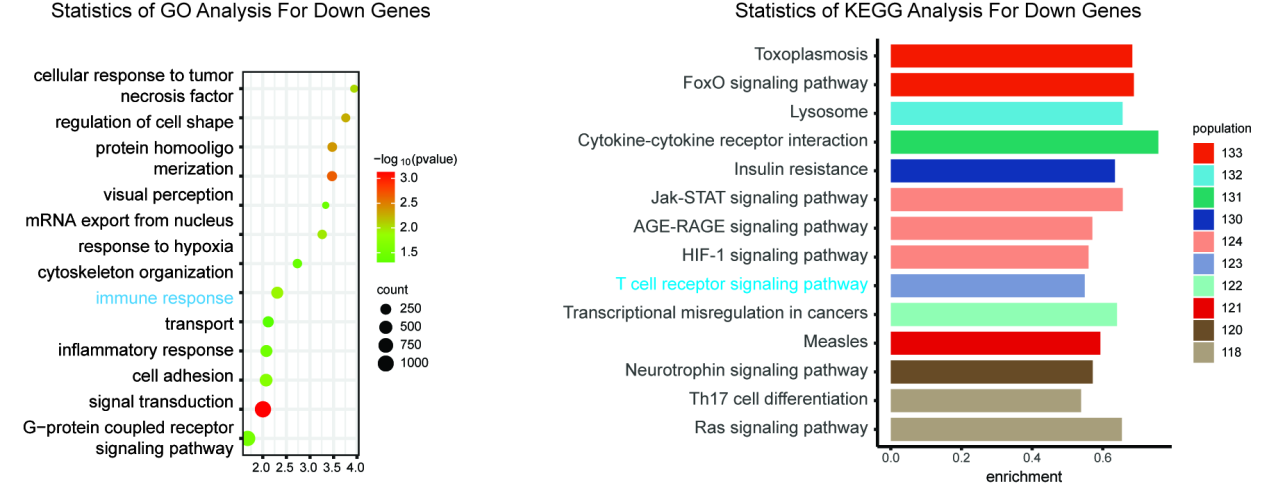


GO analysis and KEGG analysis of gene chips overexpressed in miR-375.

Supplementary Figure 3


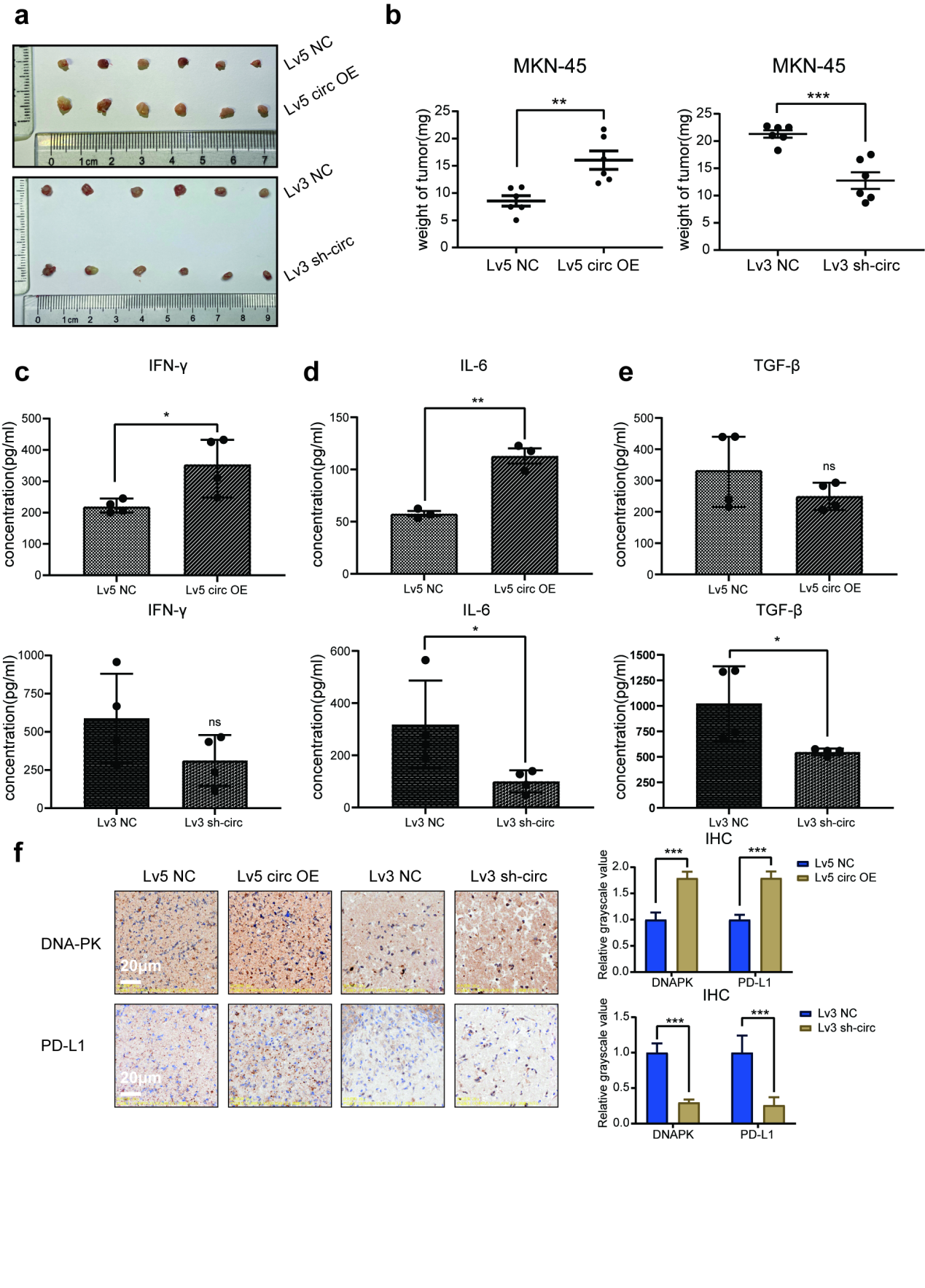


(a) Original photo of tumor in tumor bearing mice overexpressing or knocking down hsa_circ_0136666. (b) Comparison of tumor weight in tumor bearing mice, n=6. (c) Differential expression of IFN-γ in tumor regions of tumor bearing mice, n=4. (d) Differential expression of IL-6 in tumor regions of tumor bearing mice, n=4. (e) Differential expression of TGF-β in tumor regions of tumor bearing mice, n=4. (f) IHC staining of tumor sections in tumor bearing mice, five visual field ranges were randomly selected for quantification. Data are presented as the mean ± SD, Student's t-test was used. *P<0.05, **P<0.01, ***P<0.001.

Supplementary Figure 4


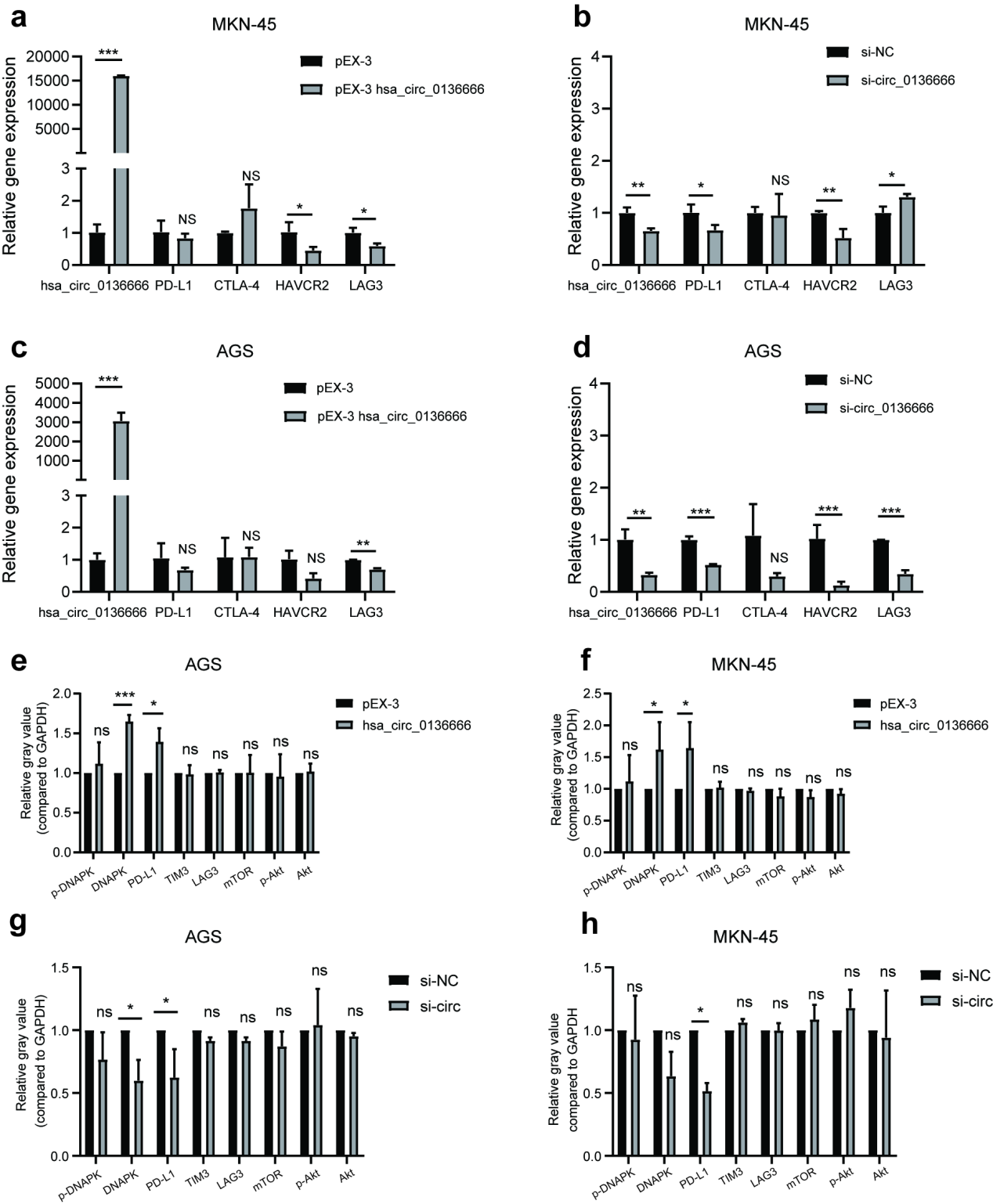


(a) QRT-PCR was used to detect mRNA levels of immune checkpoint in MKN-45 cell overexpressing hsa_circ_0136666. (b) QRT-PCR was used to detect mRNA levels of immune checkpoint in MKN-45 cell with knockdown of hsa_circ_0136666. (c) QRT-PCR was used to detect mRNA levels of immune checkpoint in AGS cell overexpressing hsa_circ_0136666. (d) QRT-PCR was used to detect mRNA levels of immune checkpoint in AGS cell with knockdown of hsa_circ_0136666. Data are presented as the mean ± SD, n=3, Student's t-test was used, *P<0.05, **P<0.01, ***P<0.001. (e-h) Quantification diagram of Western blot in Fig 2l-m. *P<0.05, ***P<0.001.

Supplementary Figure 5


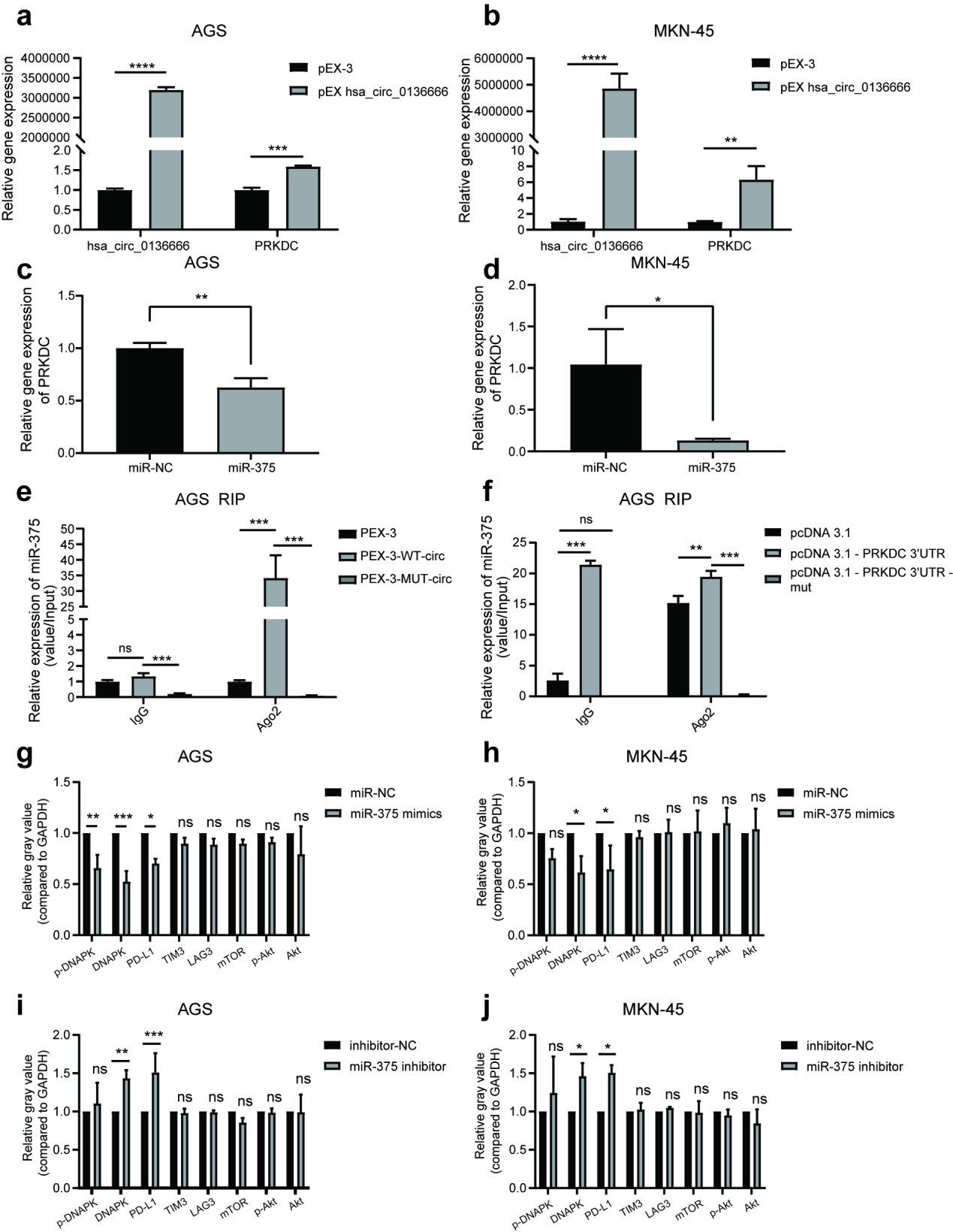


(a) QRT-PCR detection was used to detect a positive correlation between hsa_circ_0136666 and PRKDC expression in AGS cell. (b) QRT-PCR detection was used to detect a positive correlation between hsa_circ_0136666 and PRKDC expression in MKN-45 cell. (c) QRT-PCR detection was used to detect a negative correlation between miR-375 and PRKDC expression in AGS cell. (d) QRT-PCR detection was used to detect a negative correlation between miR-375 and PRKDC expression in MKN-45 cell. (e) RIP experiment was used to verify that hsa_circ_0136666 exerts sponge function. (f) RIP experiment was used to verify the combination of miR-375 and PRKDC. Data are presented as the mean ± SD, n=3, Student's t-test was used, *P<0.05, **P<0.01, ***P<0.001,****P<0.0001. (g-j) Quantification diagram of Western blot in Fig 5f, *P<0.05, **P<0.01, ***P<0.001.

Supplementary Figure 6


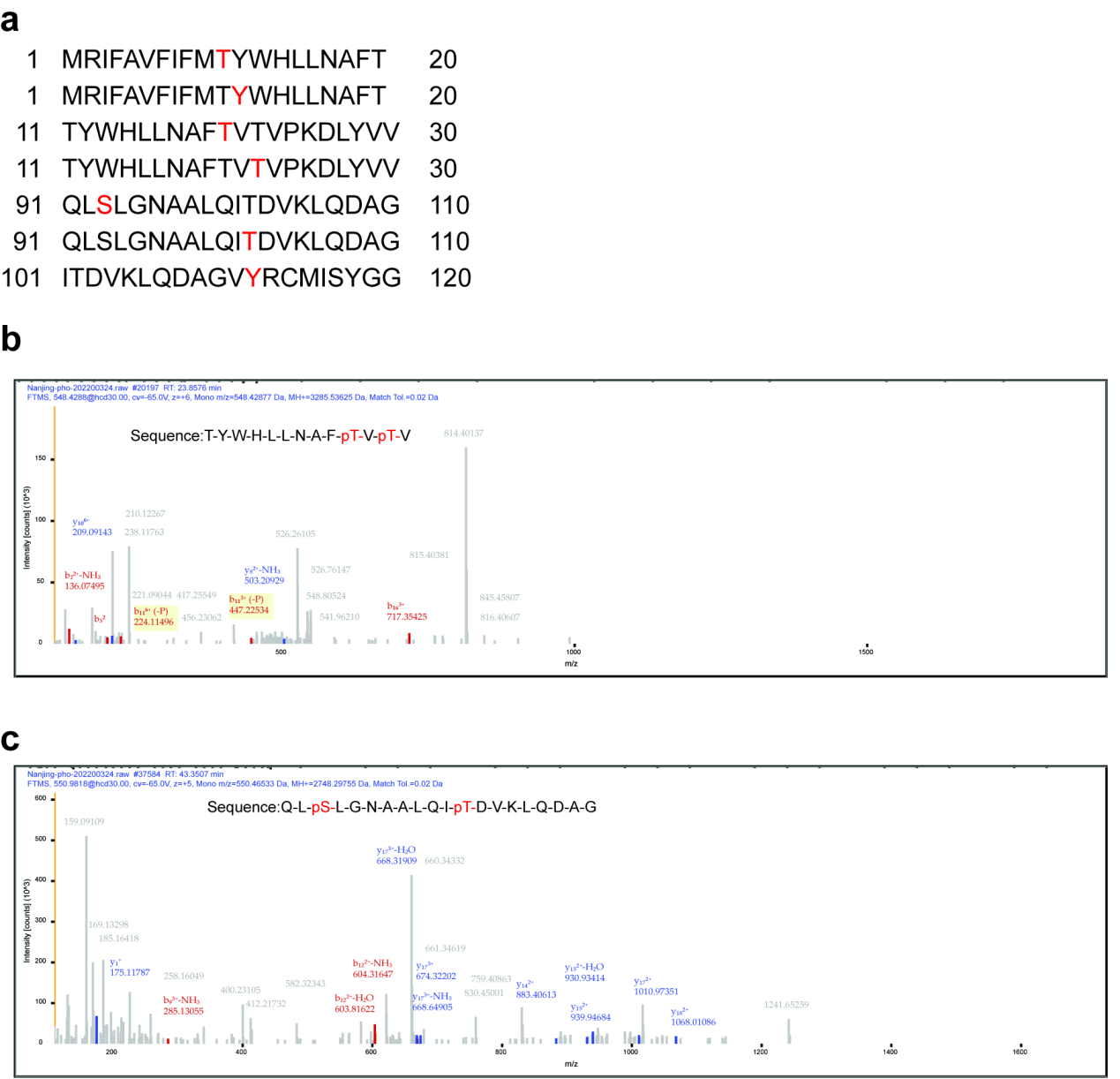


(a) Biomass spectrometry has been implemented to detect 7 possible phosphorylation sites. (b) T20T22 phosphorylated PD-L1 mass spectrometry chart. (c) S93T102 phosphorylated PD-L1 mass spectrometry chart.

Supplementary Figure 7


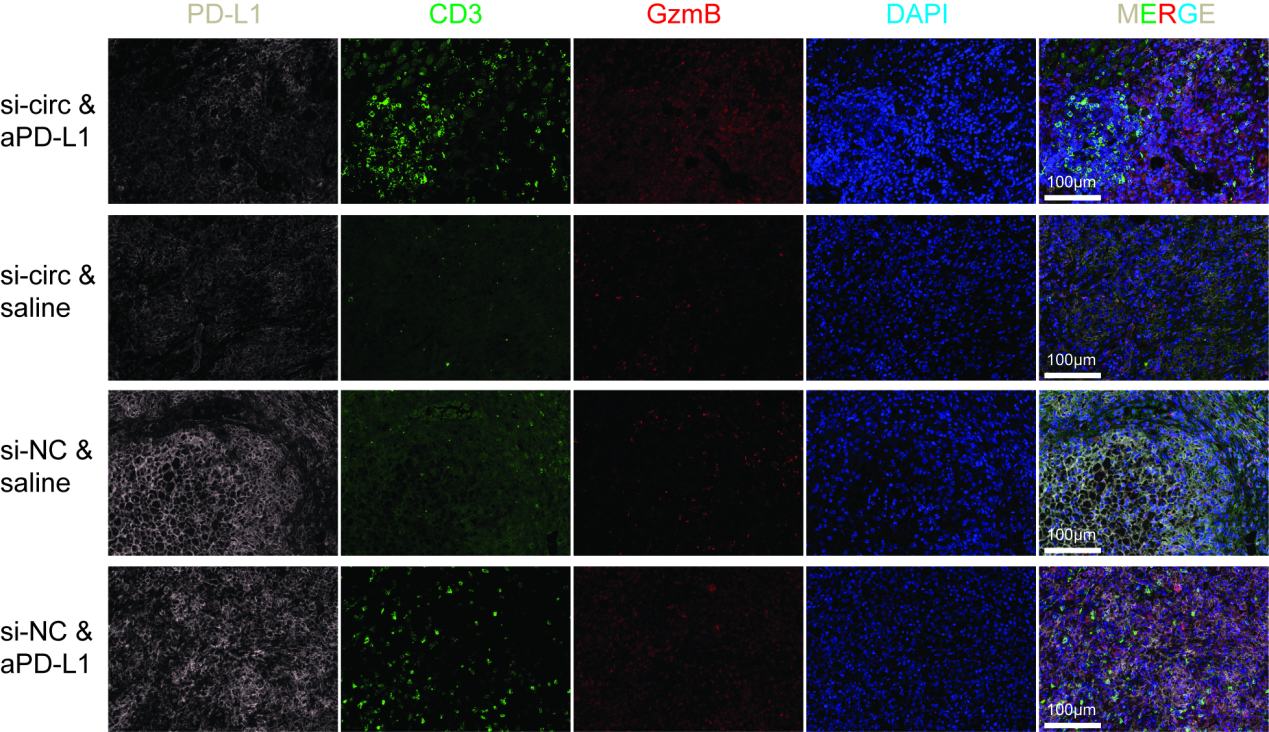


Immunofluorescence staining of tumor slices from tumor bearing mice was performed to detect immune indicators and checkpoint proteins.
